# Supplementary material for: Plug-and-play metabolic transducers expand the chemical detection space of cell-free biosensors
Source: Nat Commun. 2019 Apr 12;10:1697. doi: 10.1038/s41467-019-09722-9 (PMC6461607; doi:10.1038/s41467-019-09722-9)
Supplement: Supplementary file 1 — Description of Additional Supplementary Files [file 41467_2019_9722_MOESM1_ESM.pdf]

Title: Supplementary Data 1:
Description: List of biomarkers extracted from the HMDB database, the effectors that can directly detect those biomarkers, and the biomarkers that can be transformed via a metabolic reaction into a detectable molecules, along with the associated metabolic reaction and corresponding enzymes.

Title: Supplementary Data 2:
Description: List of metabolites that can be transformed into benzoate through a metabolic transducer, along with the metabolic reactions and the associated enzymes.
