# Supplementary material for: Plug-and-play metabolic transducers expand the chemical detection space of cell-free biosensors
Source: Nat Commun. 2019 Apr 12;10:1697. doi: 10.1038/s41467-019-09722-9 (PMC6461607; doi:10.1038/s41467-019-09722-9)
Supplement: Supplementary file 2 — Supplementary Information [file 41467_2019_9722_MOESM2_ESM.pdf]

Supplementary Information for:

Plug-and-Play Metabolic Transducers Expand the Chemical Detection Space of Cell-Free Biosensors

Voyvodic et al.

This file contains:

- **Supplementary Figure 1**: Modeling titration of transcription factor and reporter plasmids.
- **Supplementary Figure 2**: Time course of the benzoic acid biosensor response to varying concentrations of inducer.
- **Supplementary Figure 3**: Modeling metabolic transducer behavior for HipO and CocE.
- **Supplementary Figure 4**: Superfolder-GFP expression with J23101 and pBEST promoter (OR2-OR1-Pr).
- **Supplementary Figure 5**: Model-predicted shift in HipO concentration for peak biosensor signal at high concentrations of TF plasmid and inducer.
- **Supplementary Figure 6**: Time course of the hippuric acid biosensor response to varying concentrations of inducer.
- **Supplementary Figure 7**: Time course of the cocaine biosensor response to varying concentrations of inducer.
- **Supplementary Figure 8**: Time course of the benzoic biosensor response to 1x and 0.1x beverages.
- **Supplementary Figure 9**: Interference of 0.1x and 1x beverages on cell-free reaction with constitutive sfGFP plasmid.
- **Supplementary Figure 10**: Hill plot fit of a standard gradient of benzoic acid to calibrate sensor.
- **Supplementary Figure 11**: Interference of human urine on cell-free reaction with constitutive sfGFP plasmid.
- **Supplementary Figure 12**: Hill plot fit of a standard gradient of hippuric acid to calibrate sensor.
- **Supplementary Figure 13**: Correlation between cell-free biosensor and LC-MS measurements of endogenous hippuric acid levels in human urine.
- **Supplementary Figure 14**: Detection of cocaine spiked into clinical urine samples with sfGFP output module.
- **Supplementary Figure 15**: Cell-free reactions accumulate autofluorescent products in the GFP channel even in the absence of DNA.

- **Supplementary Figure 16**: Use of firefly luciferase as an output module enhances benzoic acid sensor fold change.
- **Supplementary Figure 17**: Comparison of benzoic acid and cocaine biosensor expression in response to urinary cocaine gradient.
- **Supplementary Table 1:** Fluorescence results from calibration of TF and reporter plasmids.
- **Supplementary Table 2:** Fluorescence results from calibration of HipO and CocE metabolic transducer plasmids
- **Supplementary Table 3:** Benzoate concentration in commercial beverages determined from three replicates of our cell-free biosensor and LC-MS.
- **Supplementary Table 4:** Benzoic acid sensor shows minimal activation in response to human urine without HipO metabolic transducer.
- **Supplementary Table 5:** Endogenous hippuric acid concentration in human urine samples determined from three replicates of our cell-free biosensor and LC-MS.
- **Supplementary Note 1:** SensiPath Metabolic Space Analysis
- **Supplementary Note 2:** Mathematical Model of Cell-Free Biosensors
- **Supplementary Note 3:** Mathematical-Model Derivation Appendix
- **Supplementary References**

Additional Supplementary Data files:

- **Supplementary Dataset 1:** List of biomarkers extracted from the HMDB database, the effectors that can directly detect those biomarkers, and the biomarkers that can be transformed via a metabolic reaction into a detectable molecules, along with the associated metabolic reaction and corresponding enzymes.
- **Supplementary Dataset 2:** List of metabolites that can be transformed into benzoate through a metabolic transducer, along with the metabolic reactions and the associated enzymes.
- **Supplementary Dataset 3:** Source data
- **Supplementary Dataset 4-10:** DNA sequences for plasmids used in this paper. All plasmids will be available from Addgene.

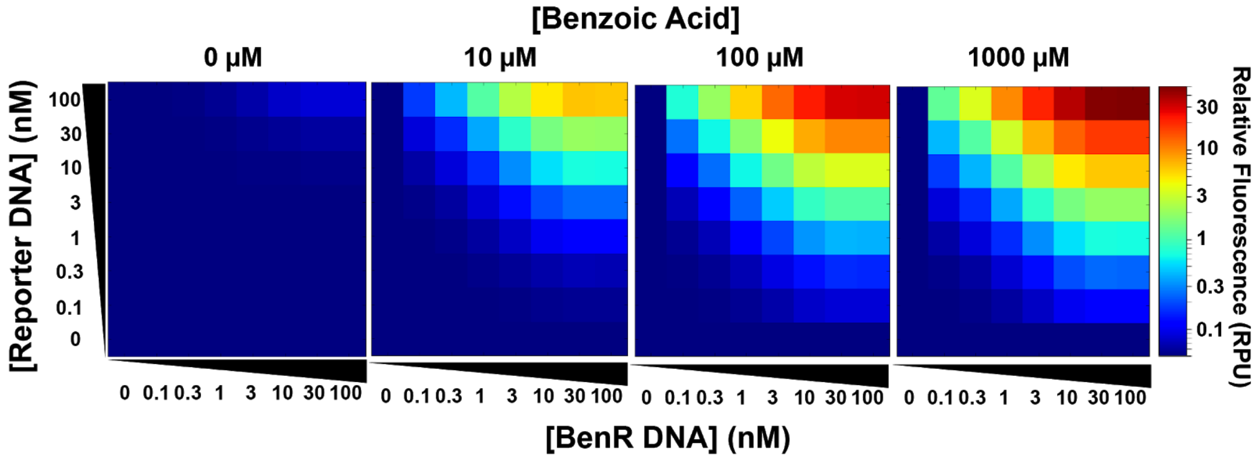

Supplementary Figure 1: Modeling titration of transcription factor and reporter plasmids. Conditions for reporter and BenR DNA concentrations used in **Figure 2** were modeled using ordinary differential equations to capture qualitative trends in the data. Simulations were rescaled to use the same scale as data. The heatmap represents GFP model signal after four hours.

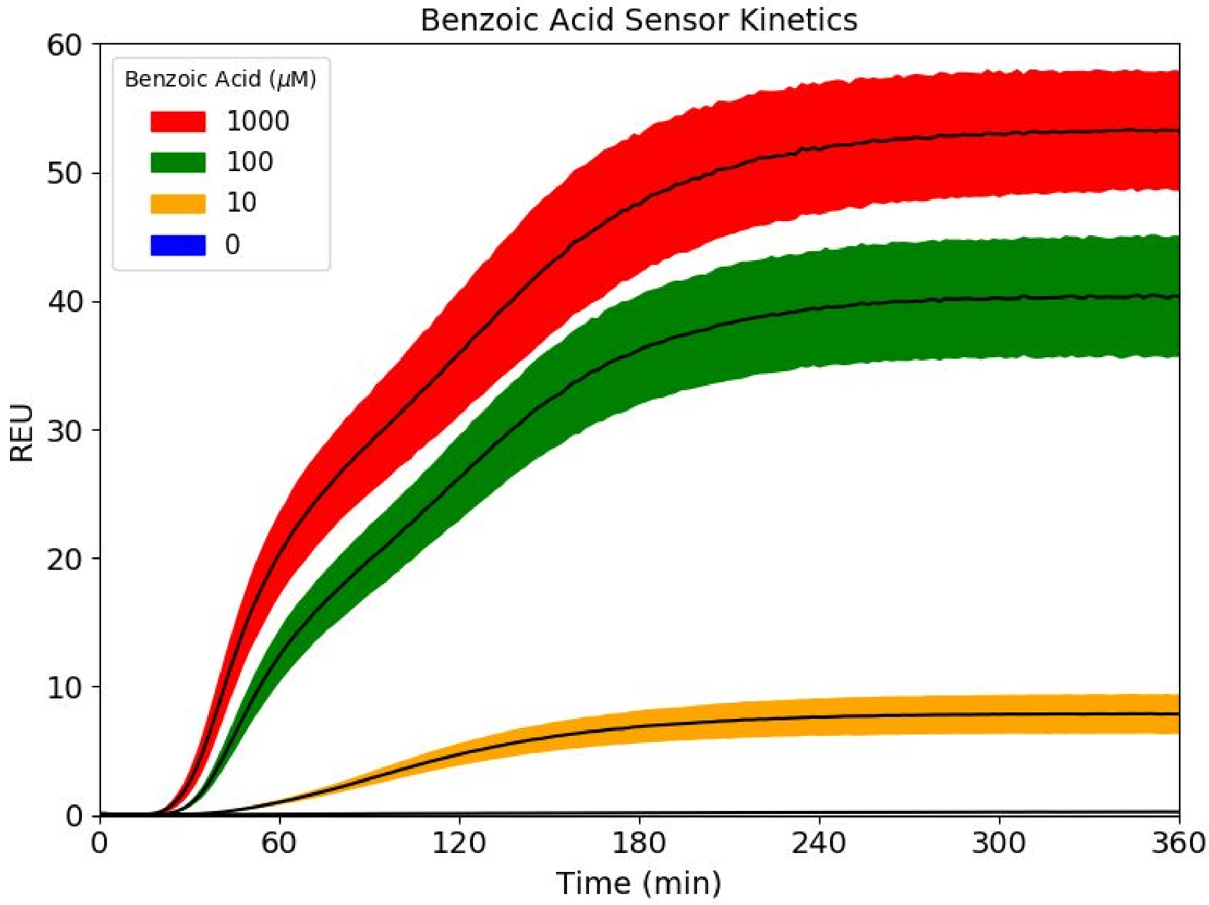

Supplementary Figure 2: Time course of the benzoic acid biosensor response to varying concentrations of inducer. Kinetics of optimized benzoic acid sensor at 37°C, where the TF plasmid concentration was 30 nM and the reporter plasmid concentration was 100 nM. Data are the average, with standard deviation, of three technical repeats from three experiments performed on three different days and all fluorescence values have relative expression units (REU) compared to the four hour level for 100 pM of a strong, constitutive sfGFP-producing plasmid. Fold change measurements were taken from the four hour time point.

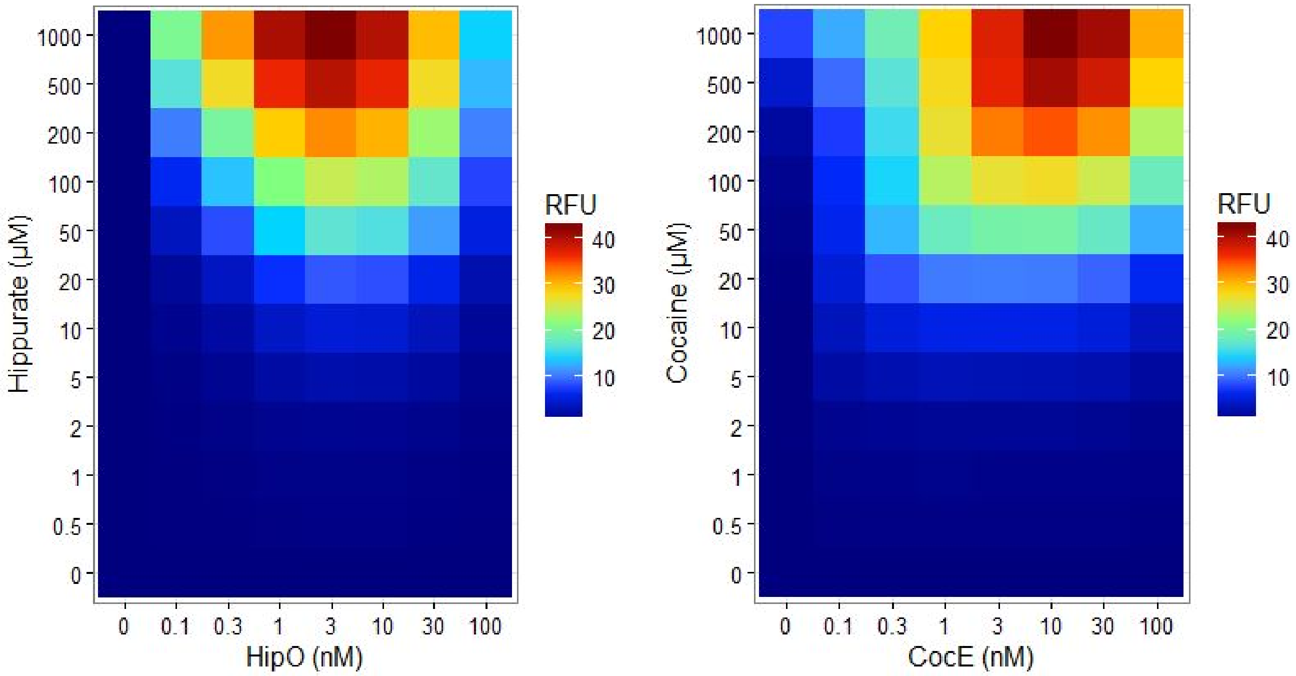

Supplementary Figure 3: Modeling metabolic transducer behavior for HipO and CocE. Hippurate or cocaine can be detected using different metabolic tranducers. Conditions for inducer and DNA concentrations used in **Figure 3** were modeled using ordinary differential equations to capture qualitative trends in the data. Simulations were rescaled to use the same scale as data. The heatmap represents GFP model signal after four hours.

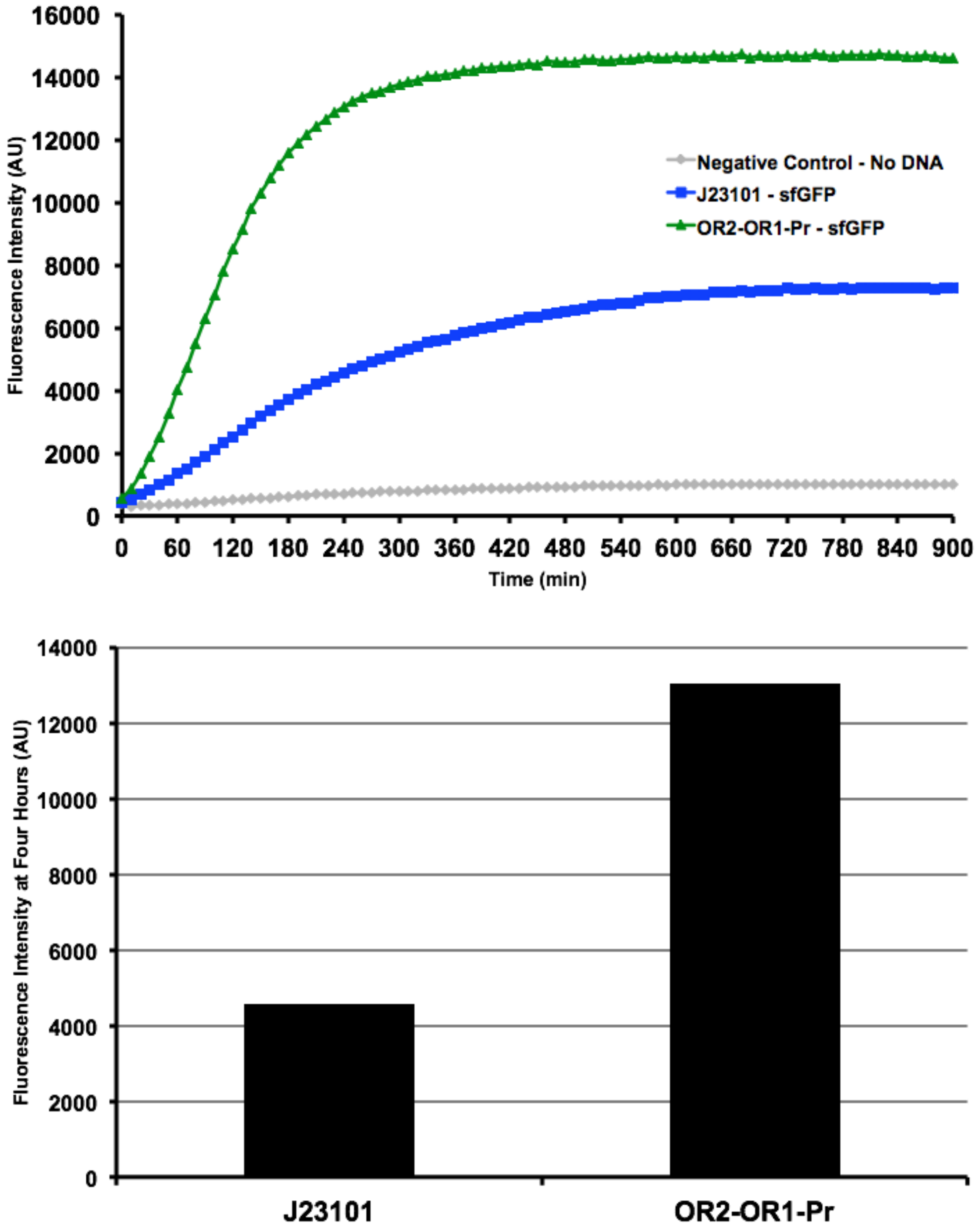

Supplementary Figure 4: Superfolder-GFP expression with J23101 and pBEST promoter (OR2-OR1-Pr). Expression levels of J23101 and OR2-OR1-Pr promoters were compared in a cell-free reaction to provide comparative strength data for our computer model. Reactions were conducted at 6.5 ng/μL at 37°C for fifteen hours and data at the four hour time point showed that J23101 is approximately three times weaker than OR2-OR1-Pr in our cell-free system.

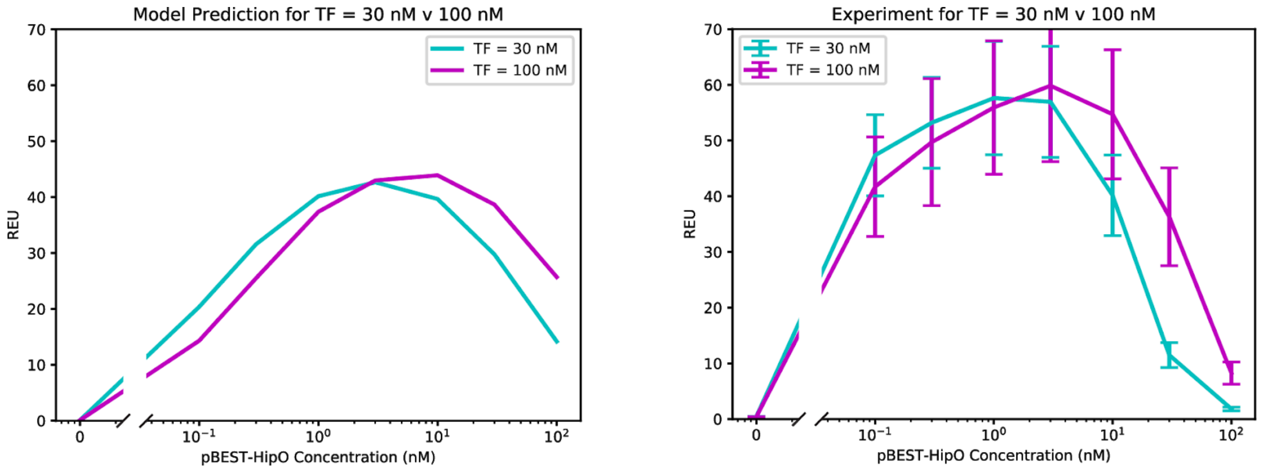

Supplementary Figure 5: Model-predicted shift in HipO concentration for peak biosensor signal at high concentrations of TF plasmid and inducer. Increasing TF plasmid concentration results in a right-shift of HipO plasmid concentration for optimal performance. Left panel: Model calculations for sfGFP output for a range of pBEST-HipO concentrations for TF plasmid concentrations at 30 nM and 100 nM. Right panel: Experimental results to examine if the same right-shift could be seen experimentally. Results are the mean from three experiments on three different days and error bars represent the standard deviation. For all experiments and model calculations, reporter plasmid concentration was fixed at 100 nM and a hippurate inducer concentration of 1000 µM was used. All fluorescence values have relative expression units (REU) compared to the four hour level for 100 pM of a strong, constitutive sfGFP-producing plasmid.

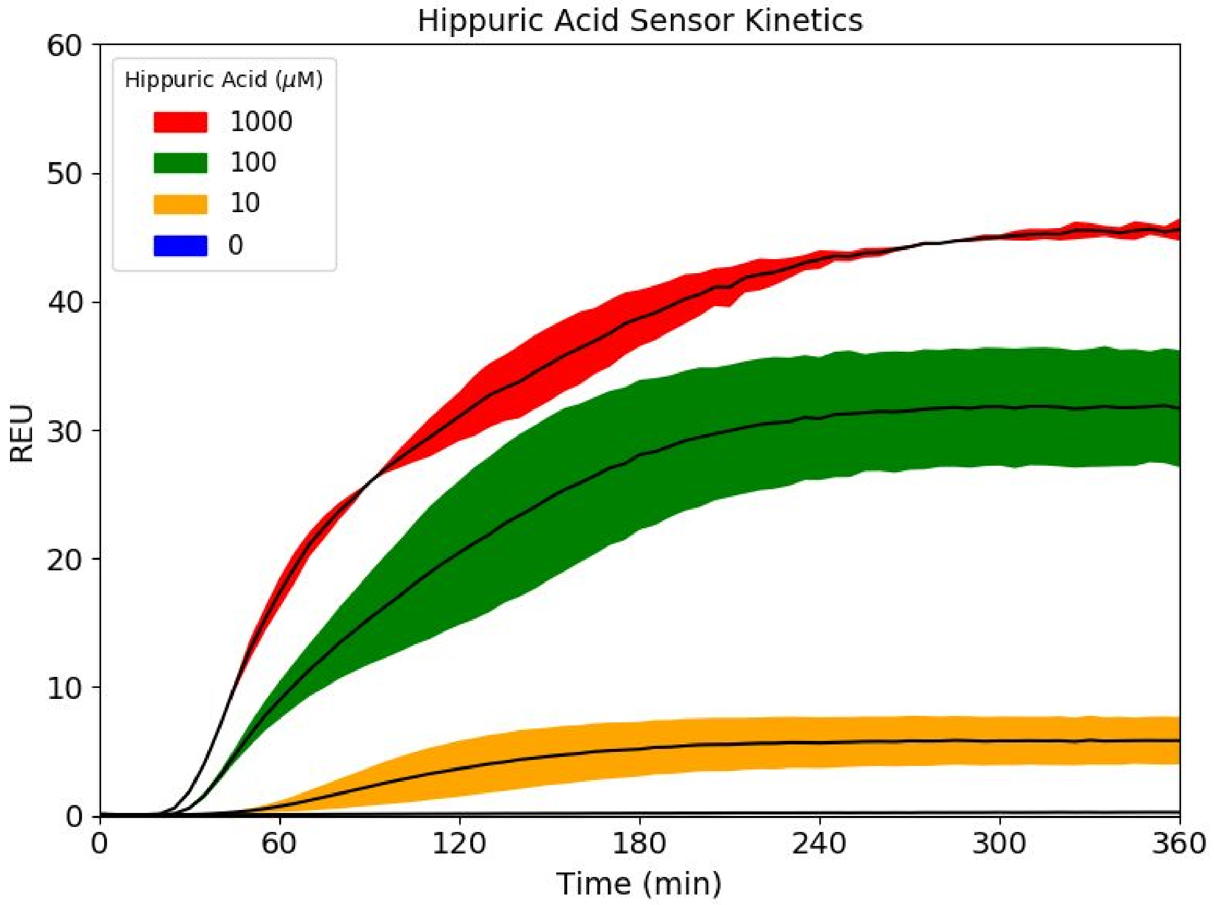

Supplementary Figure 6: Time course of the hippuric acid biosensor response to varying concentrations of inducer. Kinetics of optimized hippuric acid sensor at 37°C, where the HipO plasmid concentration was 3 nM and the TF and reporter plasmids were maintained at the same concentrations as the optimized benzoic acid sensor (30 nM and 100 nM, respectively). Data are the average, with standard deviation, of three experiments performed on three different days and all fluorescence values have relative expression units (REU) compared to the four hour level for 100 pM of a strong, constitutive sfGFP-producing plasmid. Fold change measurements were taken from the four hour time point.

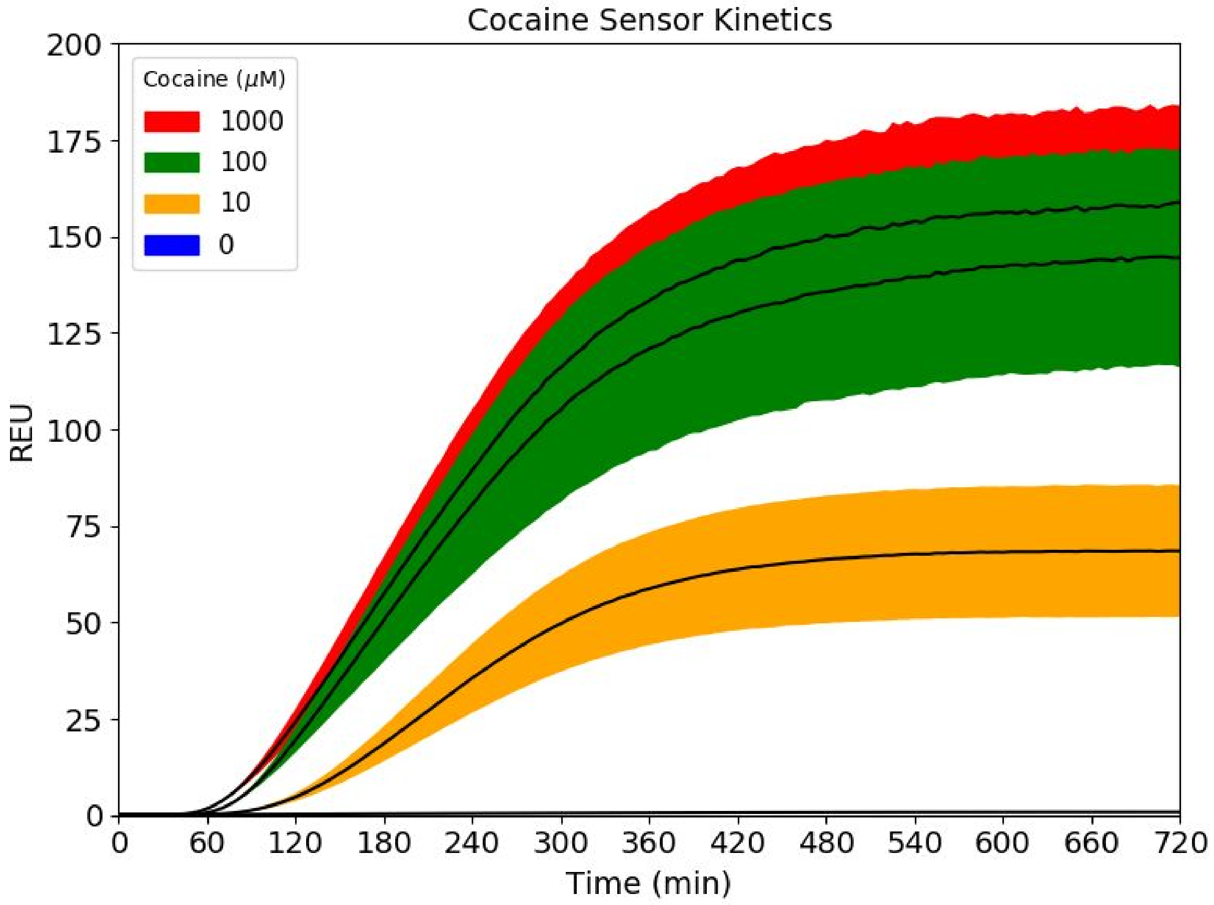

Supplementary Figure 7: Time course of the cocaine biosensor response to varying concentrations of inducer. Kinetics of optimized cocaine biosensor at 30°C, in which the CocE plasmid concentration was 10 nM and the TF and reporter plasmids were maintained at the same concentrations as the optimized benzoic acid sensor (30 nM and 100 nM, respectively). Data are the average, with standard deviation, of three experiments performed on three different days and all fluorescence values have relative expression units (REU) compared to the four hour level for 100 pM of a strong, constitutive sfGFP-producing plasmid. Fold change measurements were taken from the four hour time point.

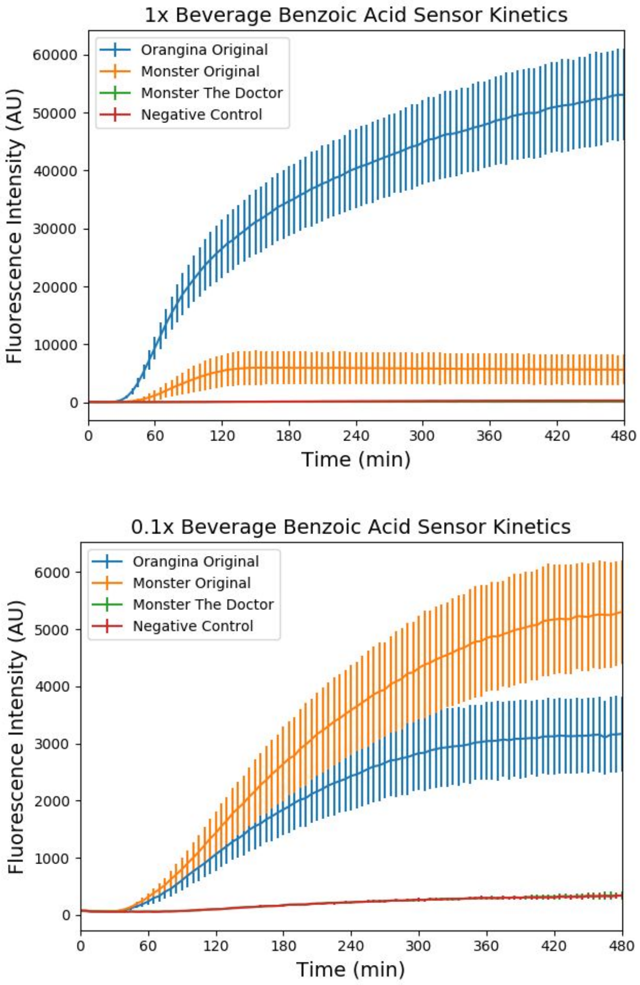

Supplementary Figure 8: Time course of the benzoic biosensor response to 1x and 0.1x beverages. Kinetics of sfGFP expression at 37°C using our optimized benzoic acid biosensor to detect benzoates in commercial beverages. The top panel depicts kinetics in response to addition of 2 µL of unaltered beverage to a 20 µL cell-free reaction. The bottom panel depicts kinetics after the samples were first diluted 1:10 in water before being added to the reaction. 'Orangina Original' and 'Monster Original' include sodium benzoate and benzoic acid, respectively, in their list of ingredients. 'Monster The Doctor' lists no benzoates in the ingredients. Water was used in place of the beverage for the negative control. Data depict the mean of three experiments conducted on three different days and error bars represent the standard deviation. Fluorescence intensity y-axis scale was adjusted for the weaker signal dilution experiment to enable adequate visualization of the kinetics.

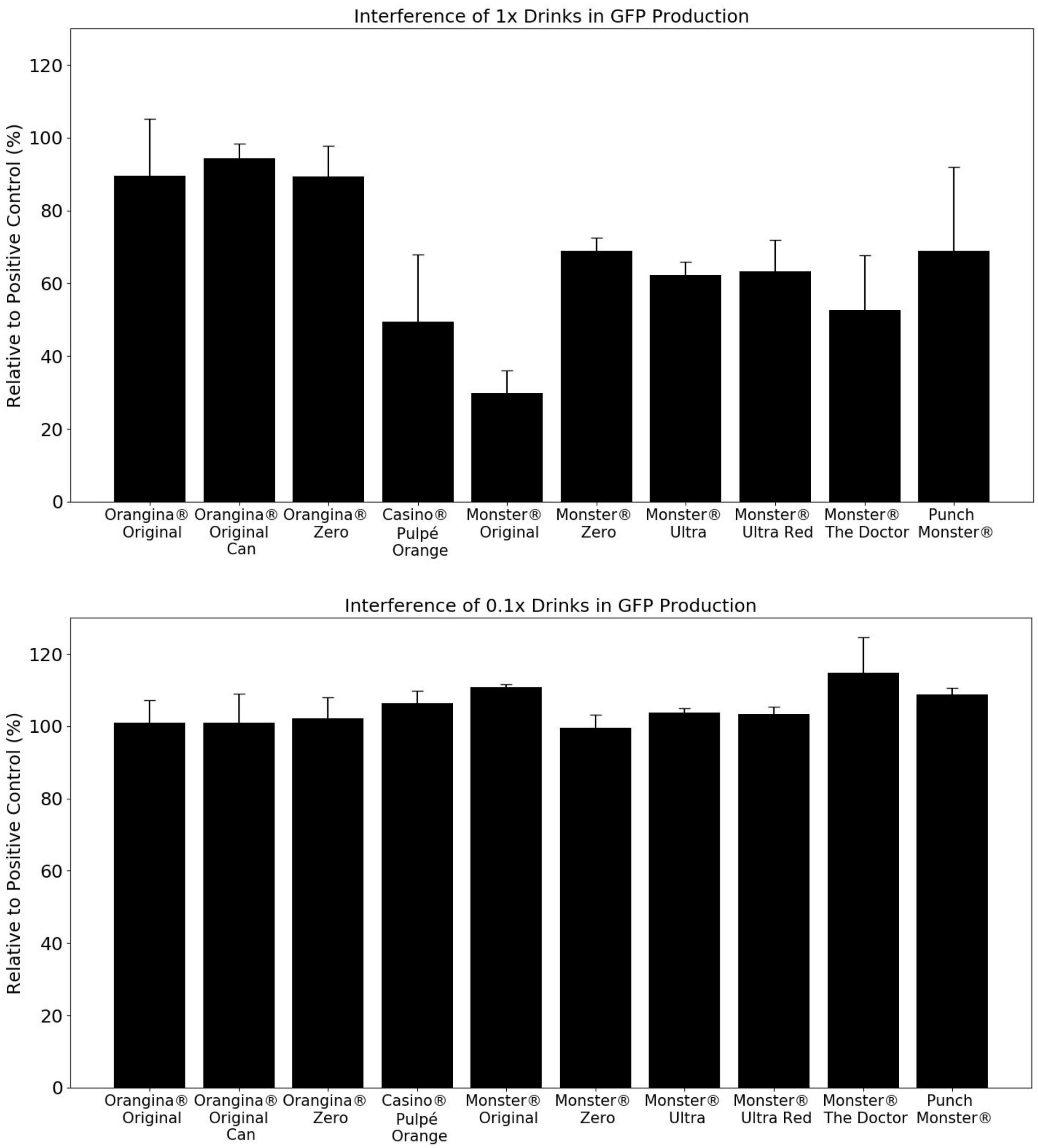

Supplementary Figure 9: Interference of 0.1x and 1x beverages on cell-free reaction with constitutive sfGFP plasmid. Ten-fold dilution of inducing beverage in water greatly reduces their interference in cell-free reactions. 2 µL of either 1x (top panel) or 0.1x (bottom panel) beverages were added to 20 µL cell-free reactions containing 10 nM of the strong constitutive GFP plasmid pBEAST-sfGFP. Fluorescence intensities at four hours were normalized to a negative control containing water instead of the commercial beverage. Data are mean values from three experiments on three different days and error bars represent the standard deviation.

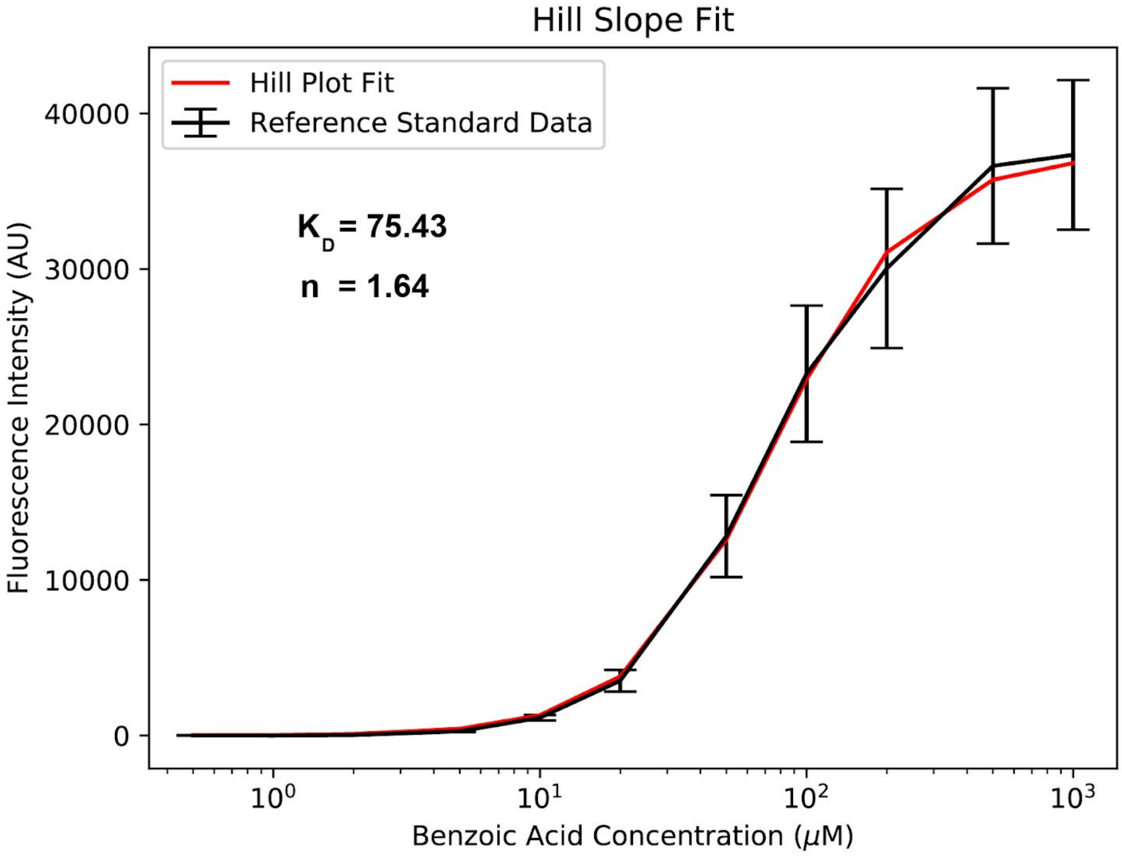

Supplementary Figure 10: Hill plot fit of a standard gradient of benzoic acid to calibrate sensor. A standard gradient of benzoic acid concentration was added to our optimized benzoic acid sensor at 37°C for four hours. The fluorescence intensity values were fit to a Hill plot function in order to convert fluorescence measurements of benzoates in beverages into sample concentration. The data are the mean of three experiments on three different days and error bars represent the standard deviation.

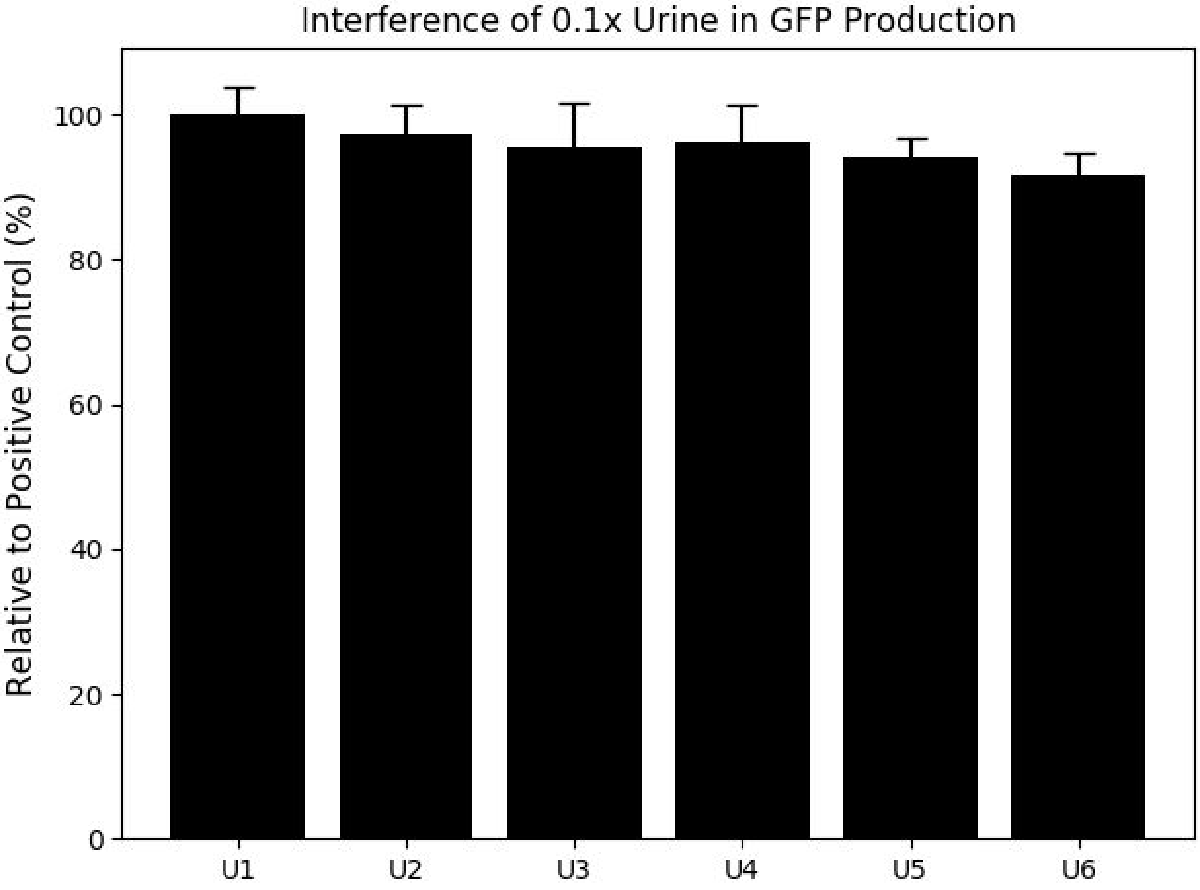

Supplementary Figure 11: Interference of human urine on cell-free reaction with constitutive sfGFP plasmid. Ten-fold dilution in urine in the presence of an RNase inhibitor minimizes interference of human urine on cell-free production. Urine samples from six patients (U1-U6) were diluted 1:10 in water and 2 μL were added to 20 μL cell-free reactions (1% final concentration) containing 10 nM of the strong constitutive GFP plasmid pBEAST-sfGFP and 0.8 U/μL of a murine RNase inhibitor. Fluorescence intensities at four hours were normalized to a negative control containing water instead of urine. Data are mean values from three experiments on three different days and error bars represent the standard deviation.

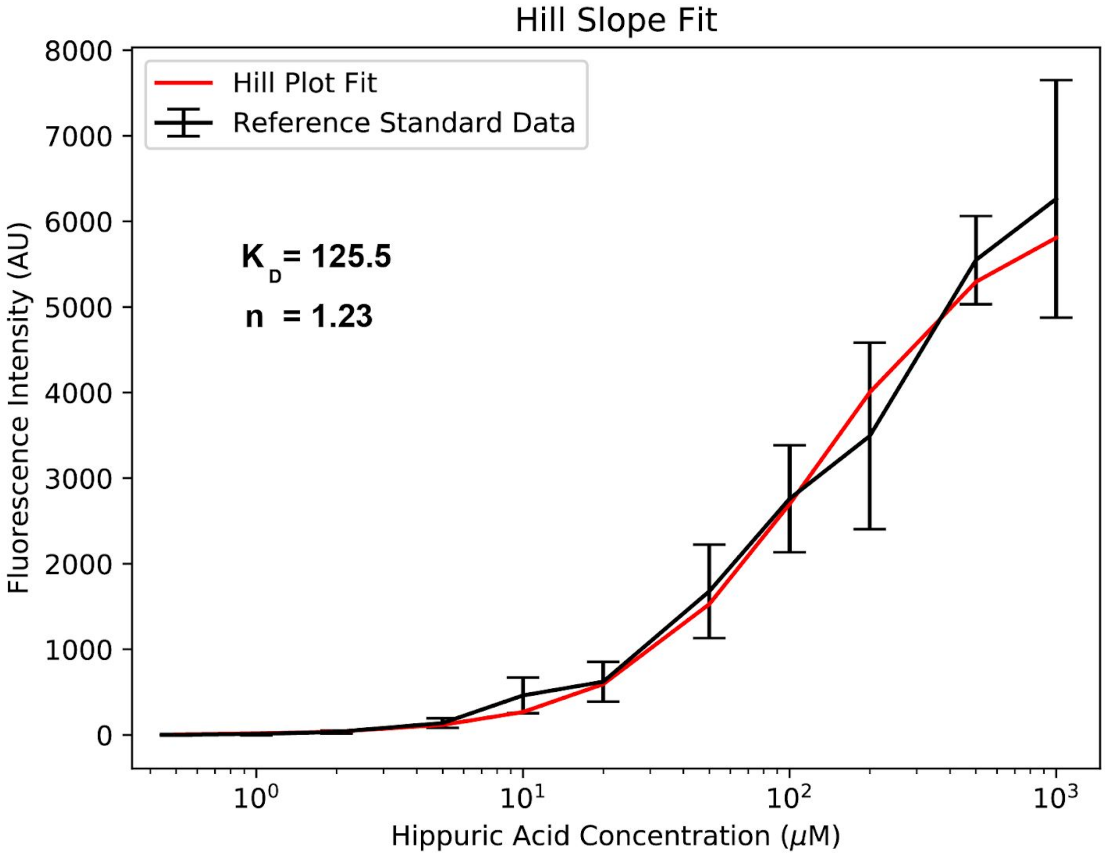

Supplementary Figure 12: Hill plot fit of a standard gradient of hippuric acid to calibrate sensor. A standard gradient of hippuric acid concentration was added to our optimized hippuric acid sensor with 0.8 U/μL of a murine RNase inhibitor at 37°C for four hours. The fluorescence intensity values were fit to a Hill plot function in order to convert fluorescence measurements of hippuric acid in urine samples into sample concentration. The data are the mean of three experiments on three different days and error bars represent the standard deviation.

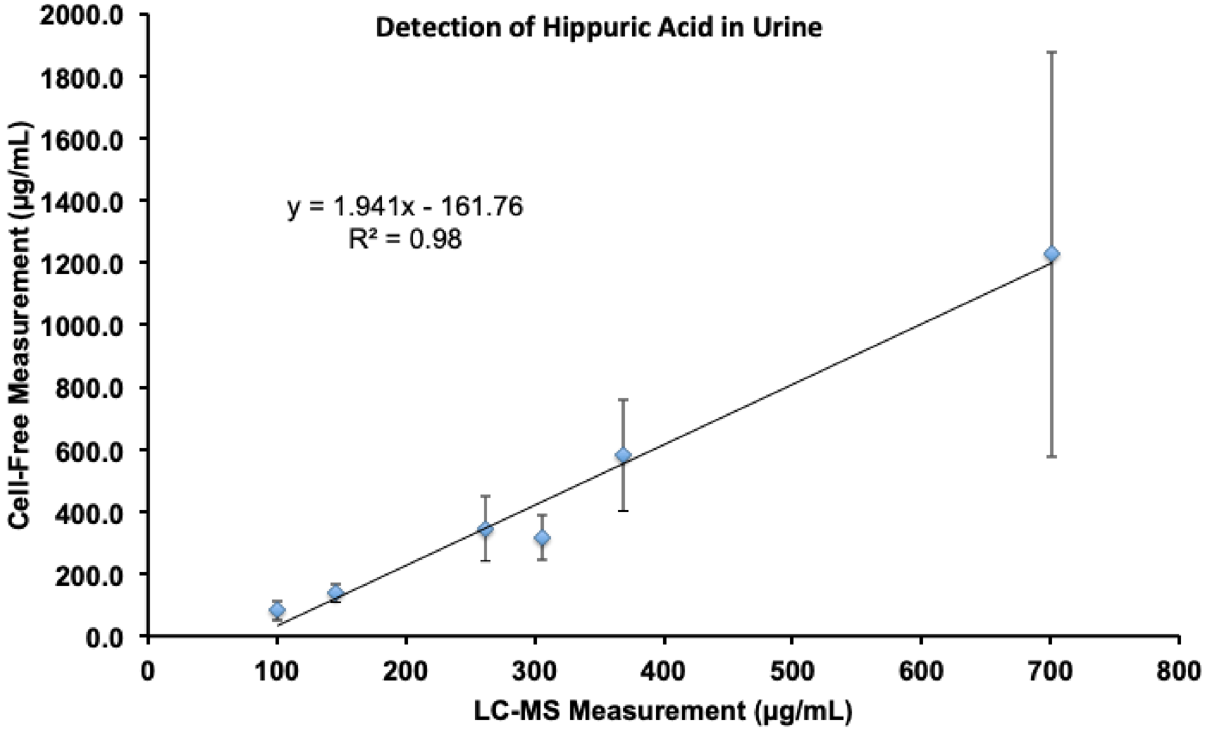

Supplementary Figure 13: Correlation between cell-free biosensor and LC-MS measurements of endogenous hippuric acid levels in human urine. Quantified cell-free biosensor values of hippuric acid measurement were determined using a Hill plot fit to our standard curve (**Supplementary Figure 12**) and cell-free data are the mean of three experiments on three different days (error bars represent standard deviation). LC-MS measurements are from a single measurement. R^2 value was calculated by a linear regression fit.

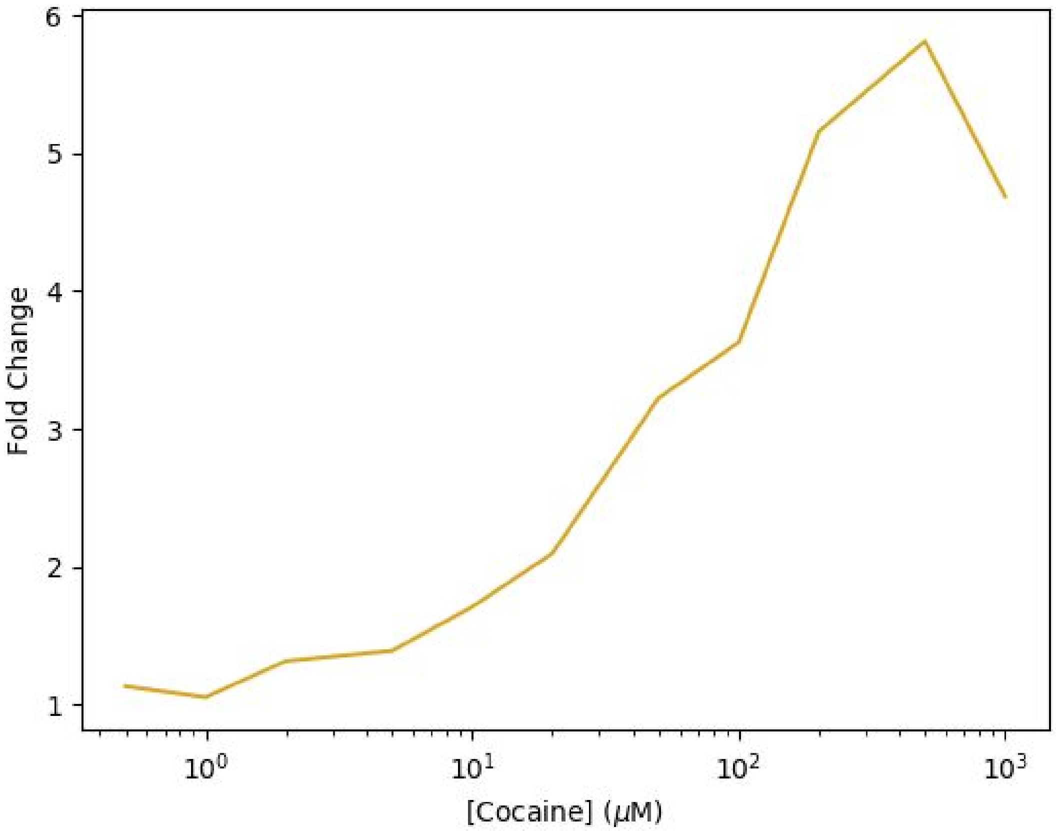

Supplementary Figure 14: Detection of cocaine spiked into clinical urine samples with sfGFP output module. A standard gradient of cocaine hydrochloride was added with 2 µL of a human urine sample to 20 µL cell-free reactions containing our optimized cocaine biosensor with 0.8 U/µL of a murine RNase inhibitor and incubated at 30°C for 8 hours. Fold change was calculated relative to the 0 µM cocaine inducer. Data are from a single pilot experiment.

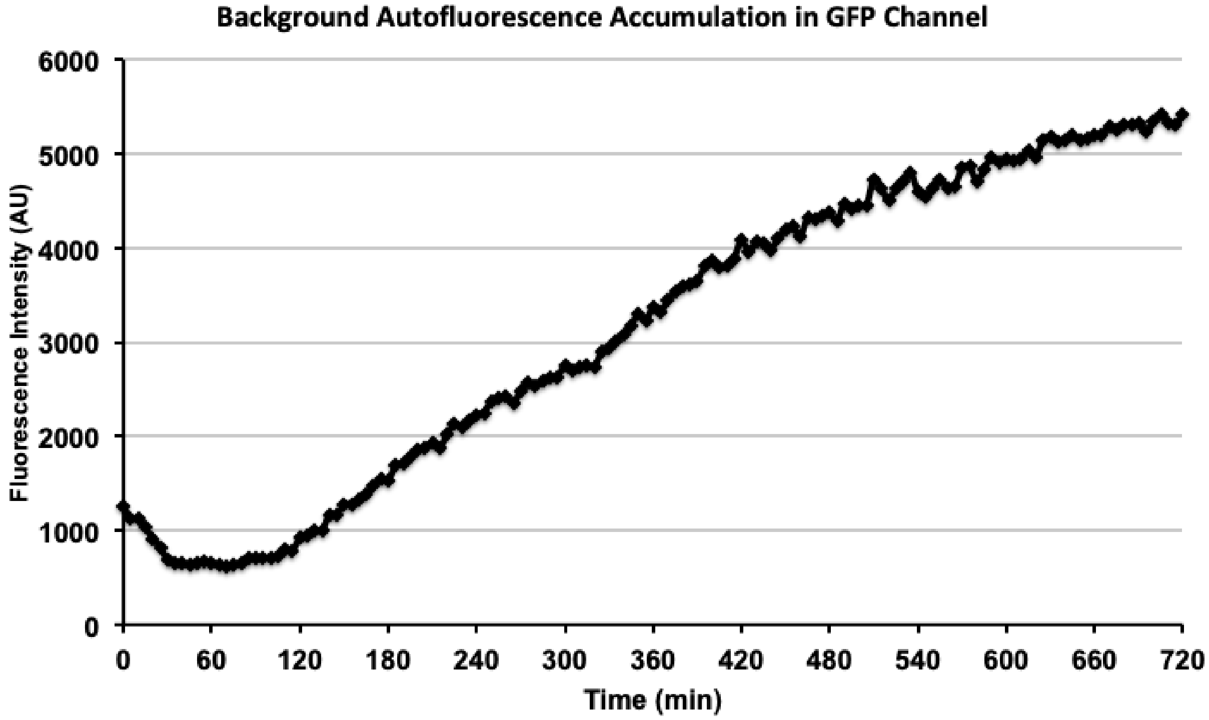

Supplementary Figure 15: Cell-free reactions accumulate autofluorescent products in the GFP channel even in the absence of DNA. Data are from one 20 µL cell-free reaction containing only buffer, extract, and water incubated at 37°C for 12 hours.

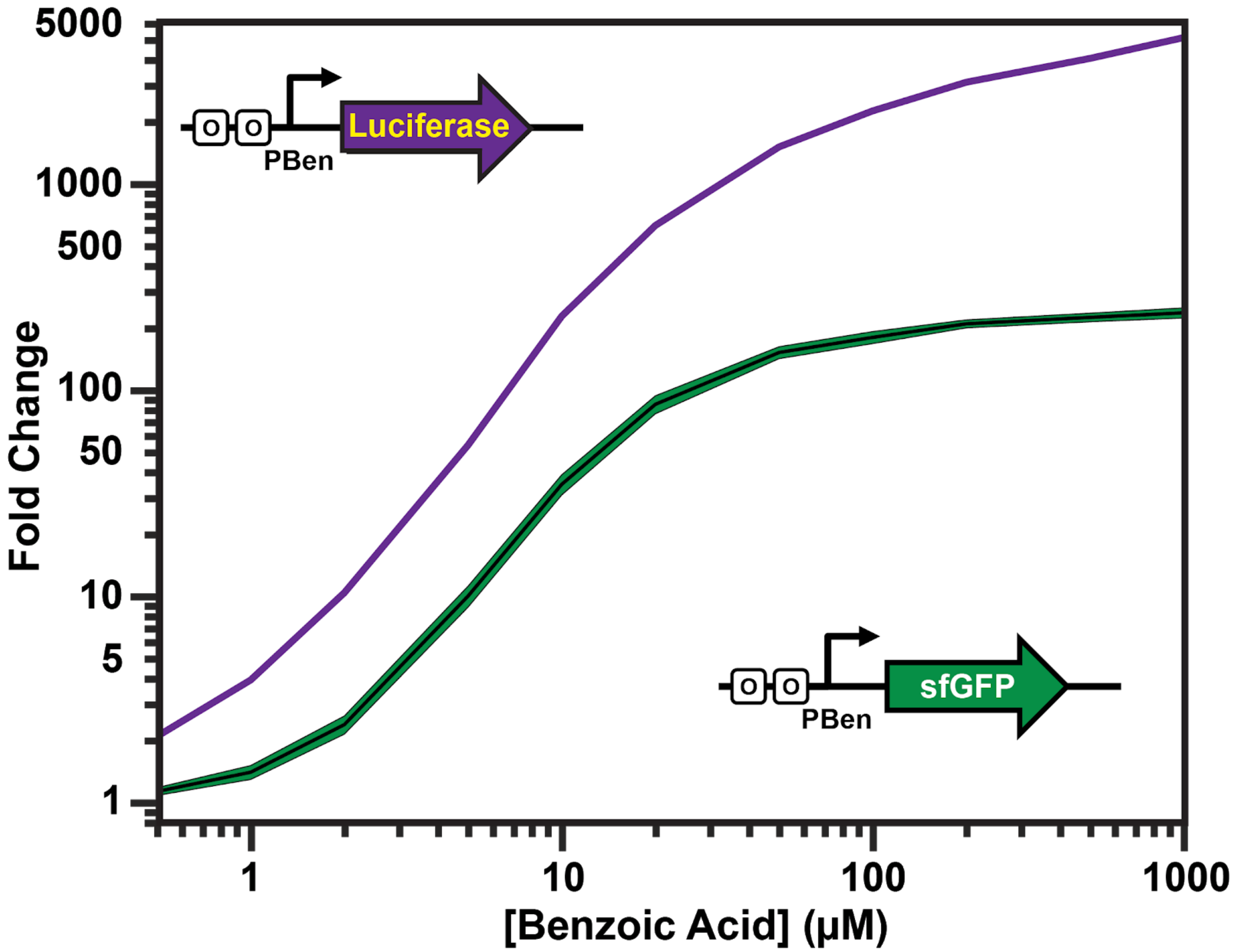

Supplementary Figure 16: Use of firefly luciferase as an output module enhances benzoic acid sensor fold change. The firefly luciferase gene was cloned under the P_{Ben} promoter and added to 20 μL cell-free reactions at the same plasmid concentrations previously used with sfGFP (TF = 30 nM; Reporter = 100 nM). Reactions were incubated at 37°C for eight hours and subsequently luciferase activity was measured on a plate reader after addition of 50 μL luciferase assay reagent. Data (purple line) was normalized to the 0 μM benzoic acid concentration and are from a single pilot experiment. Superfolder GFP curve (green line) is from Figure 2c and used as visual comparison.

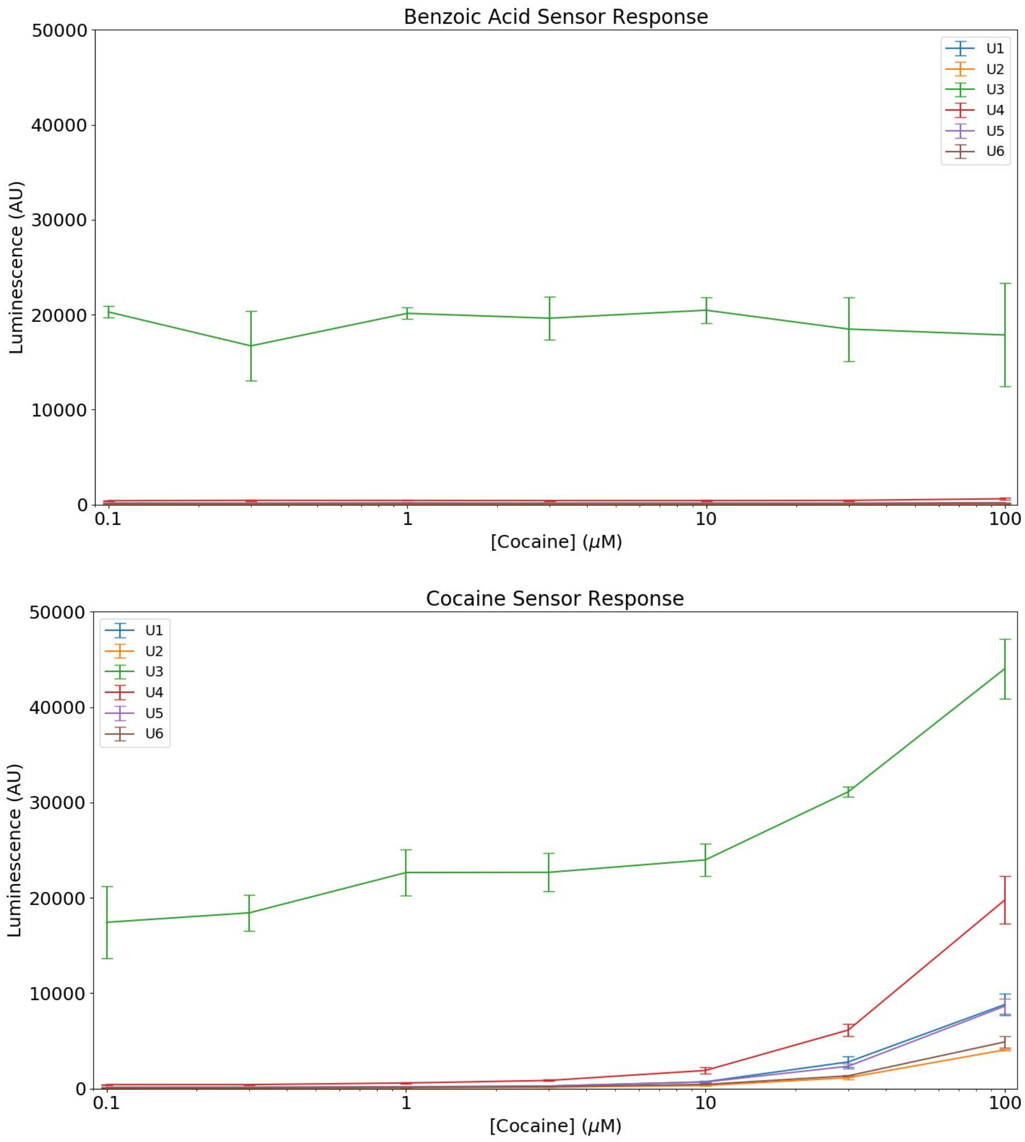

Supplementary Figure 17: Comparison of benzoic acid and cocaine biosensor expression in response to urinary cocaine gradient. A standard gradient of cocaine hydrochloride was added with 2 μL of human urine sample to 20 μL cell-free reactions containing either our optimized benzoic acid sensor or cocaine sensor with 0.8 U/μL RNase inhibitor as in **Figure 4d**. After incubate at 30ºC for eight hours, the samples were transferred to white 96-well plates and 50 μL of luciferase assay reagent was added. The plates were subsequently read on a plate reader two minutes after adding the reagent and luciferase measurements in arbitrary units (AU) are shown above for both the benzoic acid sensor (top panel) and cocaine sensor (bottom panel). Data are mean values from three experiments on three different days and error bars represent the standard deviation.

[Reporter Plasmid] (nM)	0 µM Benzoic Acid								
100	0.197 ± 0.023	0.200 ± 0.013	0.201 ± 0.018	0.195 ± 0.027	0.202 ± 0.027	0.200 ± 0.023	0.195 ± 0.034	0.177 ± 0.022	
30	0.197 ± 0.004	0.192 ± 0.010	0.187 ± 0.027	0.190 ± 0.018	0.181 ± 0.013	0.188 ± 0.023	0.193 ± 0.029	0.186 ± 0.018	
10	0.169 ± 0.011	0.170 ± 0.012	0.159 ± 0.015	0.163 ± 0.010	0.169 ± 0.014	0.187 ± 0.026	0.183 ± 0.026	0.189 ± 0.029	
3	0.152 ± 0.007	0.153 ± 0.012	0.151 ± 0.016	0.146 ± 0.007	0.155 ± 0.008	0.179 ± 0.024	0.194 ± 0.034	0.188 ± 0.024	
1	0.142 ± 0.005	0.144 ± 0.009	0.141 ± 0.014	0.140 ± 0.012	0.150 ± 0.010	0.167 ± 0.014	0.188 ± 0.029	0.177 ± 0.021	
0.3	0.143 ± 0.015	0.134 ± 0.008	0.145 ± 0.015	0.145 ± 0.014	0.151 ± 0.014	0.171 ± 0.021	0.187 ± 0.019	0.186 ± 0.027	
0.1	0.146 ± 0.005	0.148 ± 0.010	0.141 ± 0.010	0.137 ± 0.018	0.157 ± 0.022	0.165 ± 0.011	0.196 ± 0.030	0.179 ± 0.021	
0	0.150 ± 0.010	0.150 ± 0.015	0.143 ± 0.015	0.146 ± 0.014	0.147 ± 0.012	0.177 ± 0.023	0.197 ± 0.011	0.189 ± 0.018	
	0	0.1	0.3	1	3	10	30	100	[TF Plasmid] (nM)

[Reporter Plasmid] (nM)	10 µM Benzoic Acid								
100	0.196 ± 0.018	0.199 ± 0.014	0.198 ± 0.015	0.283 ± 0.036	0.714 ± 0.145	4.583 ± 0.839	8.034 ± 0.361	7.445 ± 0.734	
30	0.187 ± 0.007	0.185 ± 0.016	0.188 ± 0.010	0.241 ± 0.016	0.530 ± 0.124	3.114 ± 0.960	4.749 ± 0.609	4.894 ± 1.405	
10	0.174 ± 0.014	0.168 ± 0.009	0.161 ± 0.012	0.185 ± 0.022	0.322 ± 0.067	0.825 ± 0.204	1.892 ± 0.046	2.006 ± 0.310	
3	0.147 ± 0.004	0.143 ± 0.008	0.145 ± 0.007	0.153 ± 0.013	0.207 ± 0.022	0.352 ± 0.014	0.661 ± 0.047	0.826 ± 0.063	
1	0.145 ± 0.010	0.142 ± 0.014	0.135 ± 0.008	0.137 ± 0.010	0.166 ± 0.022	0.253 ± 0.036	0.335 ± 0.023	0.386 ± 0.030	
0.3	0.146 ± 0.013	0.142 ± 0.009	0.147 ± 0.015	0.138 ± 0.005	0.149 ± 0.018	0.180 ± 0.018	0.243 ± 0.011	0.247 ± 0.013	
0.1	0.144 ± 0.013	0.139 ± 0.011	0.134 ± 0.014	0.132 ± 0.014	0.144 ± 0.020	0.176 ± 0.006	0.216 ± 0.009	0.215 ± 0.019	
0	0.148 ± 0.006	0.141 ± 0.012	0.143 ± 0.019	0.143 ± 0.009	0.148 ± 0.017	0.186 ± 0.012	0.198 ± 0.018	0.205 ± 0.014	
	0	0.1	0.3	1	3	10	30	100	[TF Plasmid] (nM)

[Reporter Plasmid] (nM)	100 µM Benzoic Acid								
100	0.196 ± 0.017	0.230 ± 0.010	0.402 ± 0.029	2.128 ± 0.171	8.453 ± 1.804	23.268 ± 1.200	28.299 ± 4.737	28.584 ± 5.207	
30	0.188 ± 0.017	0.205 ± 0.010	0.373 ± 0.009	1.454 ± 0.190	6.325 ± 1.350	19.134 ± 1.013	23.251 ± 3.040	19.890 ± 2.750	
10	0.166 ± 0.011	0.186 ± 0.013	0.284 ± 0.004	0.913 ± 0.037	2.508 ± 0.297	4.844 ± 0.303	7.614 ± 0.214	8.724 ± 1.168	
3	0.156 ± 0.014	0.145 ± 0.012	0.174 ± 0.012	0.307 ± 0.025	0.873 ± 0.088	1.545 ± 0.087	2.110 ± 0.131	2.819 ± 0.440	
1	0.144 ± 0.003	0.143 ± 0.003	0.134 ± 0.019	0.166 ± 0.007	0.332 ± 0.035	0.588 ± 0.042	0.769 ± 0.086	0.957 ± 0.106	
0.3	0.148 ± 0.011	0.133 ± 0.006	0.136 ± 0.007	0.143 ± 0.012	0.189 ± 0.016	0.297 ± 0.032	0.329 ± 0.016	0.390 ± 0.006	
0.1	0.145 ± 0.007	0.140 ± 0.016	0.132 ± 0.011	0.137 ± 0.003	0.162 ± 0.024	0.200 ± 0.012	0.225 ± 0.030	0.258 ± 0.029	
0	0.154 ± 0.021	0.144 ± 0.014	0.141 ± 0.019	0.146 ± 0.018	0.145 ± 0.016	0.168 ± 0.017	0.184 ± 0.026	0.196 ± 0.026	
	0	0.1	0.3	1	3	10	30	100	[TF Plasmid] (nM)

[Reporter Plasmid] (nM)	1000 µM Benzoic Acid								
100	0.205 ± 0.008	0.257 ± 0.002	0.624 ± 0.085	3.329 ± 0.575	12.805 ± 0.931	27.240 ± 3.315	32.983 ± 6.468	33.464 ± 4.077	
30	0.195 ± 0.017	0.251 ± 0.012	0.553 ± 0.047	2.407 ± 0.219	9.353 ± 1.242	21.718 ± 2.330	25.349 ± 2.320	21.771 ± 4.279	
10	0.178 ± 0.005	0.192 ± 0.019	0.390 ± 0.008	1.257 ± 0.186	3.054 ± 0.262	5.401 ± 0.233	8.547 ± 0.270	10.253 ± 1.928	
3	0.163 ± 0.024	0.152 ± 0.008	0.184 ± 0.014	0.370 ± 0.023	1.103 ± 0.072	1.683 ± 0.084	2.282 ± 0.253	3.285 ± 0.778	
1	0.139 ± 0.010	0.139 ± 0.011	0.141 ± 0.010	0.171 ± 0.010	0.386 ± 0.038	0.666 ± 0.057	0.799 ± 0.086	1.087 ± 0.322	
0.3	0.141 ± 0.008	0.137 ± 0.012	0.128 ± 0.007	0.146 ± 0.007	0.194 ± 0.021	0.298 ± 0.026	0.351 ± 0.016	0.424 ± 0.034	
0.1	0.146 ± 0.020	0.128 ± 0.011	0.141 ± 0.015	0.135 ± 0.013	0.151 ± 0.004	0.205 ± 0.013	0.238 ± 0.015	0.273 ± 0.021	
0	0.137 ± 0.017	0.134 ± 0.013	0.136 ± 0.011	0.123 ± 0.012	0.137 ± 0.017	0.164 ± 0.018	0.192 ± 0.032	0.208 ± 0.024	
	0	0.1	0.3	1	3	10	30	100	[TF Plasmid] (nM)

Supplementary Table 1: Fluorescence results from calibration of TF and reporter plasmids. Values represent those in **Figure 2b** and are the mean ± standard deviation for three experiments on three different days.

[Hippuric Acid] (µM)									
1000	3.418 ± 0.937	37.338 ± 4.207	42.286 ± 2.880	44.845 ± 1.976	44.592 ± 1.666	31.485 ± 7.517	18.732 ± 3.113	2.611 ± 0.698	
500	1.823 ± 1.184	34.331 ± 3.957	37.399 ± 2.495	43.171 ± 0.853	43.814 ± 1.988	34.240 ± 2.989	15.917 ± 2.386	2.314 ± 0.686	
200	0.420 ± 0.010	32.865 ± 4.769	36.282 ± 1.553	41.395 ± 1.847	40.453 ± 3.345	29.809 ± 5.084	13.566 ± 3.224	1.808 ± 0.578	
100	0.299 ± 0.022	29.140 ± 5.284	33.189 ± 3.416	36.347 ± 3.867	34.785 ± 5.206	25.818 ± 4.628	10.590 ± 3.103	1.256 ± 0.487	
50	0.282 ± 0.022	24.886 ± 5.175	27.684 ± 4.226	28.876 ± 4.443	27.634 ± 4.623	19.913 ± 6.594	7.083 ± 2.380	0.809 ± 0.265	
20	0.267 ± 0.019	12.607 ± 3.131	14.963 ± 4.850	13.064 ± 3.845	13.148 ± 3.870	8.451 ± 3.902	2.755 ± 1.121	0.345 ± 0.100	
10	0.247 ± 0.026	6.187 ± 2.189	8.191 ± 3.457	6.260 ± 1.939	6.573 ± 1.744	3.319 ± 1.127	1.330 ± 0.549	0.251 ± 0.053	
5	0.235 ± 0.032	2.157 ± 0.793	2.129 ± 0.697	1.600 ± 0.339	2.528 ± 0.482	1.198 ± 0.365	0.456 ± 0.150	0.206 ± 0.042	
2	0.236 ± 0.031	0.534 ± 0.100	0.588 ± 0.132	0.508 ± 0.111	0.453 ± 0.090	0.363 ± 0.077	0.225 ± 0.047	0.184 ± 0.032	
1	0.244 ± 0.031	0.323 ± 0.027	0.325 ± 0.032	0.322 ± 0.045	0.296 ± 0.046	0.239 ± 0.044	0.192 ± 0.039	0.177 ± 0.036	
0.5	0.256 ± 0.028	0.283 ± 0.008	0.269 ± 0.022	0.262 ± 0.034	0.255 ± 0.044	0.213 ± 0.050	0.196 ± 0.039	0.185 ± 0.040	
0	0.264 ± 0.021	0.268 ± 0.021	0.266 ± 0.020	0.246 ± 0.039	0.244 ± 0.042	0.210 ± 0.037	0.195 ± 0.045	0.179 ± 0.040	
	0	0.1	0.3	1	3	10	30	100	[HipO Plasmid] (nM)

[Cocaine] (µM)									
1000	24.083 ± 12.948	24.127 ± 1.216	56.984 ± 6.055	80.445 ± 11.017	94.253 ± 13.664	107.991 ± 18.193	105.540 ± 15.864	89.035 ± 12.908	
500	14.616 ± 10.917	15.654 ± 1.740	54.427 ± 7.990	80.311 ± 11.604	95.633 ± 16.476	107.334 ± 18.623	102.870 ± 15.567	91.222 ± 14.606	
200	6.260 ± 4.509	8.904 ± 0.716	48.761 ± 5.815	76.948 ± 11.223	95.171 ± 16.311	106.021 ± 19.621	103.877 ± 17.949	85.047 ± 14.092	
100	5.493 ± 5.826	6.856 ± 2.948	43.217 ± 5.932	73.683 ± 11.207	86.946 ± 15.755	99.351 ± 17.942	98.562 ± 17.365	81.615 ± 14.440	
50	5.497 ± 6.513	4.600 ± 1.452	35.109 ± 6.509	69.859 ± 11.171	88.033 ± 13.621	95.244 ± 13.512	88.080 ± 11.762	64.205 ± 12.038	
20	1.956 ± 1.337	1.803 ± 0.205	24.087 ± 6.205	52.674 ± 10.508	64.834 ± 8.938	68.181 ± 11.806	65.340 ± 10.151	43.962 ± 7.367	
10	8.709 ± 11.304	1.036 ± 0.118	12.201 ± 2.314	28.312 ± 2.812	39.310 ± 7.824	40.145 ± 8.324	34.731 ± 3.889	23.756 ± 2.999	
5	3.152 ± 3.522	1.119 ± 0.026	4.878 ± 0.537	8.934 ± 1.345	13.402 ± 1.792	12.191 ± 2.943	11.799 ± 0.967	7.943 ± 0.694	
2	1.113 ± 0.760	0.733 ± 0.166	1.338 ± 0.079	1.603 ± 0.435	2.292 ± 0.348	2.230 ± 0.402	1.865 ± 0.309	1.638 ± 0.012	
1	0.502 ± 0.078	0.703 ± 0.163	0.812 ± 0.119	0.891 ± 0.150	1.017 ± 0.177	1.108 ± 0.182	0.937 ± 0.217	0.806 ± 0.097	
0.5	0.548 ± 0.132	0.591 ± 0.067	0.633 ± 0.125	0.648 ± 0.062	0.671 ± 0.077	0.803 ± 0.094	0.695 ± 0.143	0.614 ± 0.049	
0	0.495 ± 0.083	0.498 ± 0.018	0.513 ± 0.128	0.469 ± 0.056	0.475 ± 0.019	0.503 ± 0.071	0.486 ± 0.012	0.529 ± 0.025	
	0	0.1	0.3	1	3	10	30	100	[CocE Plasmid] (nM)

Supplementary Table 2: Fluorescence results from calibration of HipO and CocE metabolic transducer plasmids. Values represent those in **Figure 3a** and are the mean ± standard deviation for three experiments on three different days.

	Cell-Free Biosensor Concentration (μg/mL)				LC-MS Concentration
	Replicate 1	Replicate 2	Replicate 3	Mean ± St. Dev.	(μg/mL)
Orangina® Bottle	170.5	143.3	197.8	170.6 ± 22.3	154.23
Orangina® Can	10.3	3.4	9.6	7.7 ± 3.1	2.86
Orangina® Zero	16.6	11.8	12.3	13.6 ± 2.2	1.65
Generic Brand	18.1	13.8	10.3	14.1 ± 3.2	Not detectable
Monster® Original	304.4	172.5	217.4	231.4 ± 54.8	211.52
Monster® Absolutely Zero	147.8	139.0	193.9	160.2 ± 24.1	718.97
Monster® Ultra	172.3	150.9	154.6	159.3 ± 9.3	326.88
Monster® Ultra Red	191.1	169.0	208.4	189.5 ± 16.1	664.35
Monster® ' The Doctor'	19.0	15.6	11.0	15.2 ± 3.3	1.61
Monster® Punch	575.9	157.4	196.3	309.9 ± 188.8	315.60

Supplementary Table 3: Benzoate concentration in commercial beverages determined from three replicates of our cell-free biosensor and LC-MS. Values represent those in **Figure 4b.** Cell-free biosensor replicates are from three experiments performed on three separate days.

	Benzoic Acid Sensor Fluorescence (AU)			
Urinary Samples	Replicate 1	Replicate 2	Replicate 3	Mean ± St. Dev.
U1	148	148	144	147±2.31
U2	155	157	165	159±5.29
U3	167	193	210	190±21.7
U4	137	136	129	134±4.36
U5	150	116	131	132±17.04
U6	132	118	136	129±9.45
Negative Control	152	121	134	136±15.6

Supplementary Table 4: Benzoic acid sensor shows minimal activation in response to human urine without HipO metabolic transducer. Replicates are from three experiments performed on three separate days.

	Cell-Free Biosensor Hippuric Acid Concentration (µg/mL)				LC-MS Concentration
	Replicate 1	Replicate 2	Replicate 3	Mean ± St. Dev.	(µg/mL)
Urine 1	367.1	570.1	800.9	579.4 ± 177.2	368.90
Urine 2	97.6	167.8	152.2	139.2 ± 30.1	145.98
Urine 3	218.5	342.7	471.3	344.2 ± 103.2	261.91
Urine 4	218.5	331.3	394.3	314.7 ± 72.7	305.49
Urine 5	47.3	72.6	125.1	81.6 ± 32.4	100.47
Urine 6	697.3	840.1	2142.5	1226.6 ± 650.2	700.91

Supplementary Table 5: Endogenous hippuric acid concentration in human urine samples determined from three replicates of our cell-free biosensor and LC-MS. Values represent those in **Figure 4c**. Cell-free biosensor replicates are from three experiments performed on three separate days.

Supplementary Note 1: SensiPath Metabolic Space Analysis

In order to probe how many biosensors could be engineered using our workflow, we downloaded the HMDB database[1] as of 25/05/2018. A set of 1445 biomarkers, with a molecular weight $<$500 amu, was compiled for which at least one disease was identified (see **Supplementary Data 1**).

Next, we used the RetroPath algorithm[2] embedded in the SensiPath web server.[3] RetroPath finds metabolic pathways linking analytes (source set) to effectors (sink set), i.e. small molecules activating or inhibiting transcription factors. Taking as a sink set of 727 effectors taken from a database we recently released,[4] RetroPath was run using 20845 metabolic reaction rules extracted from MetaNetX.[5] We found that 192 out of 1445 biomarkers were effectors and could thus directly be detected by transcription factors. We also found that 1205 out of 1445 biomarkers could be transformed into 392 effectors through $\sim$80000 one-step pathways. We observed that several biomarkers could be transformed into the same effector while other biomarkers could be transformed into different effectors (see **Supplementary Data 1**). Finally, we found that $\sim$25% of biomarkers were shared by at least two diseases. Therefore, while one can develop biosensors and repurpose them for several diseases, biosensors can also be designed for a panel of biomarkers specific to a given disease. Altogether these results show a great potential for our workflow to engineer many biosensors detecting several pathological biomarkers.

We also probed to which extend our benzoate sensor could be used to detect various biomarkers. To that end, we computed how many HMDB metabolites could be connected to benzoate via RetroPath applying reverse reaction rules (computed from MetaNetX) to benzoate. We found that 64 HMDB metabolites could be transformed into benzoate via a one-step enzymatic transformation (see **Supplementary Data 2**).

Supplementary Note 2: Mathematical Model of Cell-Free Biosensors

We built a mathematical model to gain a better understanding of the behavior of our system using the metabolic transducer module. Our aim was to derive a relatively coarse-grained model that could recapitulate key behaviors observed in this dataset. The first step was to model the TF/reporter DNA assay (**Supplementary Figure 1**). We then analyzed the behaviors we wanted to reproduce in the hippurate adaptor dataset, which included: 1) increasing concentrations of hippurate led to increased signal; 2) at low HipO DNA concentrations, increasing enzyme DNA concentrations led to higher signal; and 3) at high HipO DNA concentrations, the system reaches a peak where increasing enzyme DNA concentration leads to lower signal.

Details of the full model derivation are available in **Supplementary Note 3** and scripts are available on Github at https://github.com/brsynth. Summary of the main model features are given here:

$$
\begin{aligned}
\frac{dbenzoate}{dt} &= enz * \frac{k_{cat} * inducer}{inducer + K_M} \\
\frac{dinducer}{dt} &= -enz * \frac{k_{cat} * inducer}{inducer + K_M} \\
TF_{activated} &= TF * \frac{benzoate}{benzoate + K_d^{inducer}} + 0.0005 \\
\epsilon &= \frac{TF_{activated}}{TF_{activated} + K_d^{activated}} \text{ for BenR} \\
\epsilon &= 1 \text{ for constitutive expression} \\
\frac{dmRNA}{dt} &= \gamma * n * \epsilon \frac{x}{x + \chi} * \frac{K_{tox}}{K_{tox} + tox} * \frac{R_{mRNA}}{R_{mRNA} + K_{mRNA}} - \delta * mRNA \\
\frac{dprot}{dt} &= \pi * mRNA * \frac{y}{y + k} * \frac{K_{tox}}{K_{tox} + tox} - \lambda * prot
\end{aligned}
$$

where the variables are defined as follows:

k_{cat}, K_M, enz	Enzyme Michaelis-Menten constants, enzyme concentration
TF, $TF_{activated}$	Unactivated transcription factor, transcription factor activated by benzoic acid
$K_d^{inducer}, K_d^{activated}$	Hill activation constant for the TF activation by benzoic acid/ promoter activation by TF
ϵ	Fraction of activated promoter for induced or constitutive promoters
γ, π	mRNA and protein production rates
χ, k	Affinity of the RNAP/ribosome for the promoter/RBS
x, y	Free RNAP and ribosome
tox, R_{mRNA}	Accumulated toxic by-product, available resources for mRNA production

The rest of the notation is standard, with three species for mRNA and protein considered: the enzyme, the transcription factor, and the sfGFP. Spontaneous transformation is also included in the inducer production rate for cocaine.

Increasing benzoic acid leading to increased signal was expected and we modeled this using Michaelis-Menten[6] equations for the activation of the transcription factor and of the promoter. The fact that signal was low at low TF DNA concentration and increased with increasing TF

DNA concentration meant that increasing enzyme concentration led to increased signal, which would not happen if all reactions were catalyzed on very fast time scales (i.e. the enzyme concentration would not matter). We therefore had to include enzyme kinetics in our model. At high DNA concentrations, resource competition effects meant that too many resources were diverted towards enzyme production instead of GFP production, which led to a decrease in signal. We also decided, as we know these effects exist in cell-free systems, to include resource depletion and production of toxic byproducts that would inhibit reactions in our model. For enzyme kinetics, we used the Michaelis-Menten equation[6] with parameters obtained from Brenda, whereas we used the framework developed by Gyorgy et al. for modeling resource competition, based on competition between DNA and mRNA for RNAP and ribosomes, respectively.[7] More details on the methods employed, as well as a full model derivation, are presented in **Supplementary Note 3**.

The results obtained for HipO-hippurate heatmap are presented in **Supplementary Figure 3**. No parameter fitting was performed, and minimal parameter tuning was involved, as most parameters were taken from or derived from the literature. Constants linked to resource depletion or toxic byproduct production were manually chosen so as to best capture the data, as well as ribosome or RNAP quantity. This, however, only quantitatively changed the data, but did not change the data qualitatively when parameters remained in a realistic range. Therefore, we managed to qualitatively reproduce the three effects we wanted to account for with this model, supporting our hypothesis regarding the main factors underpinning the biological effects in our HipO data.

Next, we decided to apply our model to the CocE data. We changed the enzyme kinetic parameters, as well as transcription and translation rates linked to the length of the gene; however, this failed to reproduce our experimental data, as significant signal was obtained for CocE DNA = 0.1 nM (data was very similar to HipO, despite the above-mentioned parameter changes, results not shown). We hypothesized that this was because the CocE promoter was weaker (~3x at four hours, **Supplementary Figure 4**). This shifted the peak but significant signal was still obtained for CocE DNA = 0.1 nM. However, thanks to the model, we postulated another cause due to a weaker translation initiation rate, as we were using different RBSs for the two enzymes. Using the RBS calculator, which takes context into account, we found that CocE translation initiation rate was predicted to be much slower than HipO initiation rate, which we transcribed in our model as a weaker affinity of the RBS for ribosomes.[8] Results obtained through this strategy are presented in **Supplementary Figure 3**. Using this RBS affinity change and the changed promoter strength, we managed to capture two of the three differences in the HipO and CocE datasets: signal for low CocE value starts at higher enzyme DNA concentrations (which we attribute to lower enzyme production due to a weaker promoter and putatively weaker RBS); and signal at 100 nM is higher as there are fewer resources diverted into unnecessary enzyme production (or less toxicity and resource exhaustion by unnecessary enzymes). However, we do not capture quantitative values, which could be due to the fact that measurements were performed in a different set-up or that another component our model is lacking. Moreover, the CocE experiment was performed at 30°C as it is the optimal temperature for this enzyme. Our modeling assumption was that this impacted only kinetic parameters, which is therefore included in our model. However, it might also affect the benzoic acid reporter which the model does not account for.

This shows that with our model, changing only parameters linked to the new enzyme sequence, we accurately captured the differences we aimed to capture in the two setups. Therefore, our model, without any parameter fitting and minimal parameter tuning within reasonable ranges, achieves satisfying qualitative reproduction of our data. Despite these successes, our model has limitations.

We can see that our model does not adequately capture the resource competition or exhaustion at enzyme concentration of 100 nM (although there is indeed no signal in our model if we increase the concentration of the simulated DNA to 300 nM, results not shown). To correct this limitation, including more resource exhaustion could be the answer. Moreover, although we only tried to qualitatively capture the data, the ease of explanation of CocE data after preliminary work on HipO only led us to suggest improvements that could be made to explain the data quantitatively: including GFP maturation kinetics to become fluorescent, as well as including parameters from the plate reader. However, complete quantitative modeling seems unrealistic on cell-free systems based on extracts rather than individual components, as a number of parameters still vary from batch to batch and will therefore hardly be realistically estimated for predictive modeling of the time course of the data produced on those setups without complementary experiments on each batch to determine batch-dependent relevant parameters. Qualitative predictions seem more relevant in that type of set-up at the moment. Moreover, as long as no definite hypothesis emerges as to why cell-free systems stop functioning (amino acid or nucleotide depletion, energy depletion, toxic byproduct accumulation or any other, as well as any combination of those hypotheses), different models encompassing these hypotheses will be derived mathematically, and capture some effects in the data, but no definite answer on what modeling strategy is the best can be found before this question is experimentally answered.

Model Prediction Experimental Demonstration In order to demonstrate that the predictions made by our model were trustworthy, and to test how altering the optimal TF/reporter DNA concentrations determined in the benzoic acid sensor affects the metabolic hybrid sensors, we designed a simple experimental verification. The model predicted that increasing the TF DNA concentration from our optimised concentration (30 nM) to another concentration that also gave good fold change from our initial TF reporter DNA assay (100 nM) would result in a shift of the dose-response curve of fluorescence to high transducer DNA concentration. Indeed, the unnecessary resources consumed to increase TF production would be diverted from the enzyme production that is necessary for efficient conversion of the inducer to benzoic acid. This effect is competing with the increased signal that could come from having higher TF levels, but the model predicts it to be the dominant effect, which was experimentally demonstrated using 1000 µM hippuric acid and varying the HipO concentration in two set ups, with TF concentrations either at 30 nM or 100 nM, while keeping the reporter concentration at 100 nM (**Supplementary Figure 5**). This verification leads us to have greater confidence in model predictions on effects linked to resource competition.

Supplementary Note 3: Mathematical-Model Derivation Appendix

The aim of this Supplementary Note is to present the full derivation of the model presented in 'Plug-and-Play Metabolic Transducers Expand the Chemical Detection Space of Cell-Free Biosensors'. We will first derive the full model and then explain our choices of parameters.

1 Mathematical model derivation

We will base our time model on classical models of transcription and translation and Michaelis-Menten kinetics.[6] Resource competition is mostly inspired from Gyorgy et al.,[7] except used at each time step instead of at steady-state. Resource exhaustion accounts for energy depletion and byproducts secretion. We will first present our assumptions and then expose the model as such.

1.1 Hypothesis

We will make the following assumptions:
Equilibrium of fast processes compared to transcriptional and translational elongations:

- Binding and unbinding of RNAP to DNA is on a much faster scale than elongation so considered at equilibrium
- Binding and unbinding of the transcription factor to DNA is on a much faster scale than elongation so considered at equilibrium
- Binding and unbinding of the inducer to the transcription factor is on a much faster scale than elongation so considered at equilibrium
- Binding and unbinding of ribosomes to mRNA is on a much faster scale than elongation so considered at equilibrium

Steady flow of production
We will consider the flow of RNAP and ribosomes to be at steady state. That is, we will neglect the first minutes of elongation at the start of the process before steady-state flow of production, consider that the production rate is constant and use effective production rates as explained in the subsection. Elongation itself is fast but RNAP and ribosomes are shared between processes and therefore modeling elongation and its impact on the available RNAP and ribosomes is key to modeling resource competition.

Using the same framework as Gyorgy et al.[7] for modeling resource competition, we will therefore also adopt their notations. For the sake of the reader's best understanding, we will nonetheless fully derive the model of resource competition before making further simplifications, as well as presenting our accounting of resource exhaustion and enzyme kinetics that are absent from their model.

1.2 Derivation of the resource competition model

The circuits described will consist of two types of modules: constitutively expressed ones (enzymes and the BenR transcription factor) and inducible ones (GFP), induced upon the binding by the active transcription factor u (benzoic acid/BenR complex). The promoter complex b_{ind} is formed by u binding to the empty promoter b^*_{ind} of the gene encoding the protein p_{ind} (that appears in the translation derivation). The binding of the available RNAP x can therefore form the active transcriptional complex c_{ind}, producing the mRNA m_{ind}, encoding p_{ind} at a rate γ_{ind} (encompassing all elongation reactions and accounting for the global translation rate). This

mRNA decays at a rate δ_{ind}, and all these processes encompassing transcription steps are exemplified below:

$$u + b^*_{ind} \underset{\mu^-}{\overset{\mu^+}{\rightleftharpoons}} b_{ind}, \quad b_{ind} + x \underset{\kappa^-}{\overset{\kappa^+}{\rightleftharpoons}} c_{ind}, \quad c_{ind} \xrightarrow{\gamma_{ind}} b_{ind} + x + m_{ind} \quad and \quad m_{ind} \xrightarrow{\delta_{ind}} \emptyset$$

For the constitutive expression, the model is simpler and is summarized by the following reactions:

$$b_{const} + x \underset{\kappa^-}{\overset{\kappa^+}{\rightleftharpoons}} c_{const}, \quad c_{const} \xrightarrow{\gamma_{const}} b_{const} + x + m_{const} \quad and \quad m_{const} \xrightarrow{\delta_{const}} \emptyset$$

The translation processes are identical for constitutive and inducible promoters, initiated by the binding of the ribosome y to the ribosome binding site (RBS) of the mRNA m, forming the transitionally active complex d. We consider that bound mRNA fragments cannot be degraded by RNases. Protein p is produced at a rate π encompassing elongation and production, and is degraded at a rate λ. The translation reactions are therefore:

$$m + y \underset{k^-}{\overset{k^+}{\rightleftharpoons}} d, \quad d \xrightarrow{\pi} m + y + p \quad and \quad p \xrightarrow{\lambda} \emptyset$$

The corresponding ODE system is given by equations (1) for induced proteins:

$$\begin{aligned} \frac{db_{ind}}{dt} &= (\mu^+ u b^*_{ind} - \mu^- b_{ind}) - (\kappa^+ x b_{ind} - \kappa^- c_{ind}) + \gamma c_{ind} \\ \frac{dc_{ind}}{dt} &= (\kappa^+ x b_{ind} - \kappa^- c_{ind}) - \gamma c_{ind} \\ \frac{dm_{ind}}{dt} &= \gamma c_{ind} - \delta m_{ind} - (k^+ y m_{ind} - k^- d_{ind}) + \pi d_{ind} \\ \frac{dd_{ind}}{dt} &= (k^+ y m_{ind} - k^- d_{ind}) - \pi d_{ind} \\ \frac{dp_{ind}}{dt} &= \pi d_{ind} - \lambda p_{ind} \end{aligned} \tag{1}$$

and by the following equations (2) for constitutive ones:

$$\begin{aligned} \frac{dc_{const}}{dt} &= (\kappa^+ x b_{const} - \kappa^- c_{const}) - \gamma c_{const} \\ \frac{dm_{const}}{dt} &= \gamma c_{const} - \delta m_{const} - (k^+ y m_{const} - k^- d_{const}) + \pi d_{const} \\ \frac{dd_{const}}{dt} &= (k^+ y m_{const} - k^- d_{const}) - \pi d_{const} \\ \frac{dp_{const}}{dt} &= \pi d_{const} - \lambda p_{const} \end{aligned} \tag{2}$$

1.3 RNAP and ribosome demands

1.3.1 Notations

We assume DNA concentration n_i is constant for each species. We will introduce some notations that will allow us to simplify our problem given the assumptions presented in 1.1

$$\kappa_i = \frac{\kappa_i^- + \gamma_i}{\kappa_i^+}, \quad k_i = \frac{k_i^- + \pi_i}{k_i^+}, \quad and \quad h_i = \frac{\gamma_i n_i}{\delta_i}$$

We also introduce $\mu = \frac{\mu^-}{\mu^+}$, and

$$\epsilon = \frac{\frac{u}{\mu}(1+\frac{x}{\kappa})}{1+\frac{u}{\mu}(1+\frac{x}{\kappa})}$$

As was done in Gyorgy et al.,[7] we use ϵ to describe the fraction of induced promoters for our inducible gene.

For our BenR biosensor modeling, we used

$$TF^{activated} = TF * \frac{inducer}{inducer + K_d^{inducer}} + 0.0005$$

$$\epsilon = \frac{TF^{activated}}{TF^{activated} + K_d^{activated} \times 1000}$$

The first equation represents transcription factor activation by the inducer, including some leaky activation, while the second equation represents the activation of the promoter by the activated transcription factor.

1.3.2 Simplification and resolution

Using the assumptions presented in 1.1, we can consider that

$$\begin{aligned} \frac{db}{dt} &= 0 \\ \frac{dc}{dt} &= 0 \\ \frac{dd}{dt} &= 0 \end{aligned} \tag{3}$$

This allows us to neglect binding events and consider the system to be at equilibrium for binding/unbinding events on time scales inferior to the production and degradation of mRNA and proteins. Therefore, the RNAP and ribosomes are always split between genes and mRNAs and can be solved using the same technique as in Gyorgy et al.,[7] considering resource conservation.

Using $\frac{dc}{dt} = 0$, and then $\frac{db}{dt} = 0$ we obtain:

$$\begin{aligned} \frac{dc}{dt} = 0 &\Leftrightarrow (\kappa^+ xb - \kappa^- c) - \gamma c = 0 \\ &\Leftrightarrow c = \frac{xb\kappa^+}{\kappa^+\gamma} \\ &\Leftrightarrow c = \frac{xb}{\kappa} \end{aligned} \tag{4}$$

$$\begin{aligned} \frac{db}{dt} = 0 &\Leftrightarrow (\mu^+ ub^* - \mu^- b) - \frac{dc}{dt} = 0 \\ &\Leftrightarrow (\mu^+ ub^* - \mu^- b) = 0 \\ b^* &= \frac{\mu^- b}{\mu^+ u} \end{aligned} \tag{5}$$

Using DNA conservation, ie: $n = c + b + b^*$, we have:

$$
\begin{aligned}
n &= c + b + b^* \\
&= \frac{xb}{\kappa} + b + \frac{\mu^- b}{\mu^+ u} \\
&= (\frac{x}{\kappa} + 1 + \frac{\mu^-}{\mu^+ u})b \\
b &= \frac{n}{\frac{x}{\kappa} + 1 + \frac{\mu^-}{\mu^+ u}} \\
c &= \frac{xb}{\kappa} \\
&= \frac{x}{\kappa} \quad \frac{n}{\frac{x}{\kappa} + 1 + \frac{\mu^-}{\mu^+ u}} \\
&= \frac{xn}{\kappa} \quad \frac{1}{\frac{x}{\kappa} + 1 + \frac{\mu}{u}} \\
&= \frac{xn}{\kappa} \quad \frac{u}{\mu} \quad \frac{1}{1 + \frac{u}{\mu}(1 + \frac{x}{\kappa})} \\
&= n\frac{x}{\kappa} \quad \frac{x+\kappa}{x+\kappa} \quad \frac{u}{\mu}\frac{1}{1 + \frac{u}{\mu}(1 + \frac{x}{\kappa})} \\
&= n\frac{x}{x+\kappa} \quad \frac{x+\kappa}{\kappa} \quad \frac{u}{\mu}\frac{1}{1 + \frac{u}{\mu}(1 + \frac{x}{\kappa})} \\
&= n\frac{x}{x+\kappa} \quad \frac{u}{\mu} \quad \frac{\frac{x+\kappa}{\kappa}}{1 + \frac{u}{\mu}(1 + \frac{x}{\kappa})} \\
&= n\frac{x}{x+\kappa}\epsilon
\end{aligned} \tag{6}
$$

Therefore,

$\frac{dm}{dt} = \gamma n \epsilon \frac{x}{x+\kappa} - \delta m$

For constitutive expression, derivation is much simpler, and we easily obtain $c = n\frac{x}{x+\kappa}$, or $c = n\epsilon\frac{x}{x+\kappa}$ with $\epsilon = 1$, considering all promoters are active. Using the same strategy, considering $\frac{dd}{dt} = 0$, we obtain $d = \frac{ym_f}{k}$, where m_f is the free mRNA. Considering that mRNA production and degradation is constant on the time scale of ribosome binding, and that the total amount of mRNA is m (both bound and unbound, the product of transcription from the previous steps), applying the same derivation to $m = m_f + d$ instead of $n = c + b$ leads to $d = m\frac{y}{y+k}$, and

$$\frac{dp}{dt} = \pi m \frac{y}{y+k} - \lambda p$$

Our time evolution model is therefore

$$\frac{dm}{dt} = \gamma n \epsilon \frac{x}{x+\kappa} - \delta m$$

$$\frac{dp}{dt} = \pi m \frac{y}{y+k} - \lambda p$$

1.3.3 Repartition between genes and mRNAs

This model allows us to account for resource competition by calculating the repartition of ribosomes and RNAP amongst different processes at each time step.

The explanation will be done for RNAP (x) and is similar for ribosomes (y). We consider the conservation law for RNAP:

$$X_{tot} = x + c_{GFP} + c_{enz} + c_{BenR}$$

We look for an integer x minimising the error so that

$$X_{tot} \simeq x + \epsilon \times n_{GFP} \frac{x}{x + \kappa_{GFP}} + n_{enz} \frac{x}{x + \kappa_{enz}} + n_{BenR} \frac{x}{x + \kappa_{BenR}}$$

which is the optimal RNAP repartition at this time step.

1.4 Accounting for resource depletion and toxicity

We decided to account for the exhaustion of the cell-free system in two different ways. First, we consider that there are a limited number of mRNAs that can be produced due to limited nucleotides supply or energy. This is done by multiplying transcription rates by $\frac{resources}{resources+K_d^{resource}}$ We do not consider a limit on amino acids as they are supplemented in the cell-free system, and mRNA production has been shown to stop rapidly in cell-free systems. Each mRNA produced consumes its length in nucleotides. Moreover, we consider that producing proteins also accumulates toxic byproducts, which slow down reactions for both translation and transcription, by multiplying transcription and translation rates by a function of the form $\frac{K_d^{tox}}{K_d^{tox}+ToxicProduct}$. We consider that each produced protein contributes to this effect, rather than amino acids, as we consider toxicity to be due to the fully formed proteins producing by-products or slowing down the extract. Our aim is to reproduce the exhaustion effect qualitatively.

1.5 Enzymatic steps

For modeling enzymatic steps, i.e. the conversion of the inducer (either cocaine or hippurate) into benzoic acid, we use Michaelis-Menten kinetics[6] :

$$rate = enzyme * k_{cat} \frac{substrate}{substrate + K_M}$$

2 Typical range of biochemical parameters

2.0.1 Considerations on cell-free systems

The experimental set-up (cell-free system) allows us to consider nominal DNA concentration values instead of having to consider plasmid copy number as would have to be done *in vivo*. Moreover, the rates will be derived here for *in vivo* systems and will be divided by 10 for simulations, as reactions have been shown to be slower in cell-free compared to *in vivo*[9] and an order of magnitude of difference is suggested in Underwood et al. and Niess et al.,[10].[11] Final parameters used for numerical simulations can be found at the end of the Supplemental Note.

2.0.2 Production rates

We will derive all rates as if it were *in vivo* and divide them by 10 for cell-free modeling.

According to Dennis and Bremer,[12] the mRNA chain elongation rate is ≈ 50 nucleotides per sec. The mRNA production rate γ in minutes is therefore $\gamma_{protein} = \frac{50}{length_{protein}} * 60$. Moreover, the peptide chain elongation rate is ≈ 15 amino acids per sec, which means the protein production rate π in minutes is therefore $\pi_{protein} = \frac{15}{length_{protein}} * 60$.

Protein name	Length in nucleotides	Length in amino acids	γ	π
GFP	720	240	4.2 / min	3.75 / min
BenR	954	318	3.35 / min	2.83 / min
HipO	1200	400	2.5 / min	2.25 / min
CocE	1700	560	1.76 / min	1.61 / min

In vivo transcription and translation rates

2.0.3 Degradation rates

Since the mRNA half-life is measured to be about 15 minutes in cell-free systems,[13] we use $\delta = 0.05\,/\,\mathrm{min}$. For in vivo systems, mRNA half-life is shorter, around 4 min, so we use $\delta = 0.2\,/\,\mathrm{min}$.

The protein half-life is approximately 1 hour *in vivo*.[14] As our system is purified from extract, we consider that proteases are still present and we use $\lambda = 0.0016\,/\,\mathrm{min}$ (*in vivo* rate divided by 10). Changing it affects time evolution but not the effect of DNA and inducer concentrations at 240 min that were studied in this article (results not shown).

2.0.4 Transcription and translation rates

According to Bernstein et al.,[15] there can be a transcription initiation every 5 seconds on a DNA strand. Using the fact that the mRNA chain elongation rate is $\approx$ 50 nucleotides per sec, there are, on the same DNA, at most ω RNAP, with $\omega = round(\frac{length_{protein}}{50*5}) + 1$. We will rather consider the genes to be present in $\omega * n$ numbers and being able to recruit only 1 RNAP.

In the same manner, we have to account for the fact that multiple ribosomes can be translating the same mRNA strand, but we will assume the average distance between ribosomes to be around 80 nucleotides. We then have at most χ ribosomes on a strand, where $\chi = round(\frac{length_{protein}}{80}) + 1$, and we will consider mRNA to be able to bind a single ribosome, with an effective protein production rate of $\chi * \pi$ for each mRNA.

Protein name	Length in nucleotides	ω	χ
GFP	720	4	10
BenR	954	5	13
HipO	1200	6	16
CocE	1700	8	22

Number of RNAP/ ribosomes per DNA/ mRNA strand

Protein name	χ	π	Effective *in vivo* π	Effective cell-free π
GFP	10	3.75	37.5	3.75
BenR	13	2.83	36.79	3.679
HipO	16	2.25	36	3.6
CocE	22	1.61	35.42	3.542

Effective translation rates *in vivo* and in cell-free

2.0.5 Enzymes' catalytic constants

For the two enzymes considered, CocE and HipO, the values used from BRENDA are listed in Table ??.[16] The exact values in our cell-free system may differ from the values in BRENDA as these are often measured *in vitro* and vary according to the organism the enzyme is taken from and the organism or cell-free extract it is expressed in. However, we believe they should still be accurate within an order of magnitude and we expect small changes to have minimal effect on simulation end results due to their fast kinetics related to the other system components. Moreover, despite their possible disadvantages, we prefer using literature values when possible so as to leave a minimum number of parameters free.

**Protein name**	$k_{cat}, 1/min$	K_M in µM
HipO	5880	764
CocE	3060	5.7

Enzymes' catalytic constants

2.0.6 Handling of RBS and DNA binding

Using the same order of magnitude for RNAP binding constants as Gyorgy et al.,[7] we used: κ_{GFP} = $100\,\mathrm{nM}$; κ_{HipO} = κ_{BenR} = $3000\,\mathrm{nM}$, as these are expressed constitutively under the same promoter. Since CocE was on a promoter that was weaker than that of HipO (See **Supplementary Figure 3**), we used κ_{CocE} = $4500\,\mathrm{nM}$.

Following the same reasoning, we use $k_{GFP} = 1\,\mathrm{\mu M}$, and k_{BenR} = k_{HipO} = $10\,\mathrm{\mu M}$. Moreover, using the RBS calculator,[17] we found that using gene context and the RBS, initiation of CocE is slower than initiation of HipO. Knowing that the RBS calculator is more trustworthy for trends than qualitative values, we implemented that using k_{CocE} = $30\,\mathrm{\mu M}$, i.e.: less efficient in binding ribosomes, since initial elongation rate does not appear in our modeling framework. This value was chosen at it recapitulates our data well.

2.1 Numerical simulations

2.1.1 Parameters

Parameters used for the final simulations are presented at the end of this Supplemental Note. A constant value of 0.05 is added to account for background on all data points.

3 Computational methods

3.0.1 Software tools

All scripts were done in R (version 3.2.3),[18] using RStudio as an integrated development environment (version 0.99.903).[19] The ODE solver used is ode from the deSolve package (version 1.14).[20] For visualization, packages reshape2[21] and ggplot2[22] are used.

3.0.2 Availability

Scripts are available on Github at `https://github.com/brsynth`.

Parameter	Value	Unit
κ_{GFP}	100	nM
κ_{BenR}	3000	nM
κ_{HipO}	3000	nM
κ_{CocE}	4500	nM
γ_{GFP}	0.42	min^{-1}
γ_{BenR}	0.335	min^{-1}
γ_{HipO}	0.25	min^{-1}
γ_{CocE}	0.176	min^{-1}
k_{GFP}	1	M
k_{BenR}	10	M
k_{HipO}	10	M
k_{CocE}	30	M
π_{GFP}	3.75	min^{-1}
π_{BenR}	3.679	min^{-1}
π_{HipO}	3.6	min^{-1}
π_{CocE}	3.542	min^{-1}
$length^{mRNA}_{GFP}$	720	nucleotides
$length^{mRNA}_{BenR}$	954	nucleotides
$length^{mRNA}_{HipO}$	1200	nucleotides
$length^{mRNA}_{CocE}$	1700	nucleotides
k^{HipO}_{cat}	5880	min^{-1}
k^{CocE}_{cat}	3060	min^{-1}
k^{HipO}_{M}	764	mM
k^{CocE}_{M}	5.7	mM
$Spontaneoushydrolisation^{HipO}$	0	M
$Spontaneoushydrolisation^{CocE}$	0.0001	M
ω_{GFP}	4	No unit
ω_{BenR}	5	No unit
ω_{HipO}	6	No unit
ω_{CocE}	8	No unit
$K^{inducer}_{d}$	100	M
$K^{activated}_{d}$	50	M
δ	0.05	min^{-1}
λ	0.0016	min^{-1}
n_{GFP}	100	nM
n_{BenR}	30	nM
X	30	nM
Y	30	nM
K^{tox}_{d}	100	nM
K^{mRNA}_{d}	10	nucleotides
$Initial^{resource}$	10000000	nucleotides

Numerical parameters used during simulations

Supplementary References

[1] Wishart, D. S. *et al.* HMDB 4.0: the human metabolome database for 2018. *Nucleic Acids Res.* **46**, D608–D617 (2018).

[2] Delépine, B., Duigou, T., Carbonell, P. & Faulon, J.-L. RetroPath2.0: A retrosynthesis workflow for metabolic engineers. *Metab. Eng.* **45**, 158–170 (2018).

[3] Delpine, B., Libis, V., Carbonell, P. & Faulon, J.-L. SensiPath: computer-aided design of sensing-enabling metabolic pathways. *Nucleic Acids Res.* gkw305 (2016).

[4] Koch, M., Pandi, A., Delépine, B. & Faulon, J.-L. A dataset of small molecules triggering transcriptional and translational cellular responses. *Data Brief* **17**, 1374–1378 (2018).

[5] Moretti, S. *et al.* MetaNetX/MNXref–reconciliation of metabolites and biochemical reactions to bring together genome-scale metabolic networks. *Nucleic Acids Res.* **44**, D523–6 (2016).

[6] Michaelis, L. & Menten, M. L. Die kinetik der invertinwirkung. *Biochem. Z* **49**, 352 (1913).

[7] Gyorgy, A. *et al.* Isocost Lines Describe the Cellular Economy of Genetic Circuits. *Biophysical Journal* **109**, 639–646 (2015).

[8] Borujeni, A. E., Channarasappa, A. S. & Salis, H. M. Translation rate is controlled by coupled trade-offs between site accessibility , selective RNA unfolding and sliding at upstream standby sites **42**, 2646–2659 (2014).

[9] Siegal-gaskins, D., Noireaux, V. & Murray, R. M. Biomolecular resource utilization in elementary cell-free gene circuits. *American Control Conference (ACC), 2013* 1531–1536 (2013). 1307.0178.

[10] Underwood, K. A., Swartz, J. R. & Puglisi, J. D. Quantitative polysome analysis identifies limitations in bacterial cell-free protein synthesis. *Biotechnology and Bioengineering* **91**, 425–435 (2005).

[11] Nieß, A., Failmezger, J., Kuschel, M., Siemann-Herzberg, M. & Takors, R. Experimentally validated model enables debottlenecking of in vitro protein synthesis and identifies a control shift under in vivo conditions. *ACS Synthetic Biology* **6**, 1913–1921 (2017).

[12] Dennis, P. P. & Bremer, H. Modulation of Chemical Composition and Other Parameters of the Cell at Different Exponential Growth Rates. *EcoSal Plus* **3** (2008).

[13] Siegal-Gaskins, D., Tuza, Z. A., Kim, J., Noireaux, V. & Murray, R. M. Gene circuit performance characterization and resource usage in a cell-free "breadboard". *ACS Synthetic Biology* **3**, 416–425 (2014).

[14] Kramer, G. *et al.* Proteome-wide alterations in Escherichia coli translation rates upon anaerobiosis. *Molecular & cellular proteomics : MCP* **9**, 2508–16 (2010).

[15] Bernstein, J. A., Khodursky, A. B., Lin, P.-H., Lin-Chao, S. & Cohen, S. N. Global analysis of mRNA decay and abundance in Escherichia coli at single-gene resolution using two-color fluorescent DNA microarrays. *Proceedings of the National Academy of Sciences* **99**, 9697–9702 (2002).

[16] Placzek, S. *et al.* BRENDA in 2017: new perspectives and new tools in BRENDA. *Nucleic Acids Research* **45**, D380–D388 (2017).

[17] Espah Borujeni, A., Channarasappa, A. S. & Salis, H. M. Translation rate is controlled by coupled trade-offs between site accessibility, selective RNA unfolding and sliding at upstream standby sites. *Nucleic Acids Research* **42**, 2646–2659 (2014).

[18] R Core Team. *R: A Language and Environment for Statistical Computing*. R Foundation for Statistical Computing, Vienna, Austria (2016). URL `https://www.R-project.org/`.

[19] RStudio Team. *RStudio: Integrated Development Environment for R*. RStudio, Inc., Boston, MA (2016). URL `http://www.rstudio.com/`.

[20] Soetaert, K., Petzoldt, T. & Setzer, R. W. Solving differential equations in r: Package desolve. *Journal of Statistical Software* **33**, 1–25 (2010). URL `http://www.jstatsoft.org/v33/i09`.

[21] Wickham, H. Reshaping data with the reshape package. *Journal of Statistical Software* **21**, 1–20 (2007). URL `http://www.jstatsoft.org/v21/i12/`.

[22] Wickham, H. *ggplot2: Elegant Graphics for Data Analysis* (Springer-Verlag New York, 2016). URL `http://ggplot2.org`.
