# Supplementary material for: Plug-and-play metabolic transducers expand the chemical detection space of cell-free biosensors
Source: Nat Commun. 2019 Apr 12;10:1697. doi: 10.1038/s41467-019-09722-9 (PMC6461607; doi:10.1038/s41467-019-09722-9)
Supplement: Supplementary file 6 — Reporting Summary [file 41467_2019_9722_MOESM6_ESM.pdf]

nature research

Corresponding author(s): Jerome BONNET NCOMMS-18-28693A

Last updated by author(s): Jan 30, 2019

Reporting Summary

Nature Research wishes to improve the reproducibility of the work that we publish. This form provides structure for consistency and transparency in reporting. For further information on Nature Research policies, see Authors & Referees and the Editorial Policy Checklist.

Statistics

For all statistical analyses, confirm that the following items are present in the figure legend, table legend, main text, or Methods section.

n/a	Confirmed	
☐	☒	The exact sample size (n) for each experimental group/condition, given as a discrete number and unit of measurement
☐	☒	A statement on whether measurements were taken from distinct samples or whether the same sample was measured repeatedly
☒	☐	The statistical test(s) used AND whether they are one- or two-sided<br>*Only common tests should be described solely by name; describe more complex techniques in the Methods section.*
☒	☐	A description of all covariates tested
☒	☐	A description of any assumptions or corrections, such as tests of normality and adjustment for multiple comparisons
☐	☒	A full description of the statistical parameters including central tendency (e.g. means) or other basic estimates (e.g. regression coefficient) AND variation (e.g. standard deviation) or associated estimates of uncertainty (e.g. confidence intervals)
☒	☐	For null hypothesis testing, the test statistic (e.g. F, t, r) with confidence intervals, effect sizes, degrees of freedom and P value noted<br>*Give P values as exact values whenever suitable.*
☒	☐	For Bayesian analysis, information on the choice of priors and Markov chain Monte Carlo settings
☒	☐	For hierarchical and complex designs, identification of the appropriate level for tests and full reporting of outcomes
☒	☐	Estimates of effect sizes (e.g. Cohen's d, Pearson's r), indicating how they were calculated

Our web collection on statistics for biologists contains articles on many of the points above.

Software and code

Policy information about availability of computer code

Data collection	Plate reader data collected the Biotek Gen5 software
Data analysis	Excel, some custom python scripts available upon request.

For manuscripts utilizing custom algorithms or software that are central to the research but not yet described in published literature, software must be made available to editors/reviewers. We strongly encourage code deposition in a community repository (e.g. GitHub). See the Nature Research guidelines for submitting code & software for further information.

Data

Policy information about availability of data

All manuscripts must include a data availability statement. This statement should provide the following information, where applicable:

- Accession codes, unique identifiers, or web links for publicly available datasets
- A list of figures that have associated raw data
- A description of any restrictions on data availability

Source data for main text figures, along with details on models, and DNA sequences for all constructs are provided in supplementary materials. All other raw data are available from the corresponding authors on reasonable request.

Field-specific reporting

Please select the one below that is the best fit for your research. If you are not sure, read the appropriate sections before making your selection.

☒ Life sciences ☐ Behavioural & social sciences ☐ Ecological, evolutionary & environmental sciences

Life sciences study design

All studies must disclose on these points even when the disclosure is negative.

Sample size	A sample size of three was chosen for most experiments to ensure reproducibility.
Data exclusions	No data were excluded from the analyses.
Replication	Experiments were run in as three separate experiments over three different days.
Randomization	This is not relevant to our study as our independent variables were usually DNA or inducer concentrations.
Blinding	Blinding was not relevant to our study; however, the LC-MS measurements for benzoic acid and hippuric acid were performed at a separate platform facility that had not seen the values calculated by our cell-free sensor.

Reporting for specific materials, systems and methods

We require information from authors about some types of materials, experimental systems and methods used in many studies. Here, indicate whether each material, system or method listed is relevant to your study. If you are not sure if a list item applies to your research, read the appropriate section before selecting a response.

Materials & experimental systems

n/a	Involved in the study	
☒	☐	Antibodies
☒	☐	Eukaryotic cell lines
☒	☐	Palaeontology
☒	☐	Animals and other organisms
☐	☒	Human research participants
☒	☐	Clinical data

Methods

n/a	Involved in the study	
☒	☐	ChIP-seq
☒	☐	Flow cytometry
☒	☐	MRI-based neuroimaging

Human research participants

Policy information about studies involving human research participants

Population characteristics	Urine samples were obtained from patients hospitalized in Endocrinology department, but no other distinction was made regarding past medical history.
Recruitment	Patients were included after receiving a full explanation of the nature of the study and providing their written informed consent. All experimental methods were carried out in accordance with the ethical guidelines determined by the National Ministry of Health, Labour and Welfare and the Declaration of Helsinki.
Ethics oversight	This study was approved by the local ethics committee of the University Hospital of Nîmes, France: IRB number 190102

Note that full information on the approval of the study protocol must also be provided in the manuscript.
